# Dose–Response Effects of Different Exercise Modalities on Blood Pressure Control in Patients with Chronic Kidney Disease: A Systematic Review and Network Meta-Analysis

**DOI:** 10.3390/life16081243

**Published:** 2026-07-27

**Authors:** Dongze Li, Kaiming Chen, Shibo Kong, Zhengyu Gou, Xinmiao Feng, Jiezhong Wu

**Affiliations:** 1 School of Sports Science and Engineering, East China University of Science and Technology, Shanghai 200231, China; lidongze414@163.com; 2 Faculty of Sports Science, Ningbo University, Ningbo 315211, China; 3 School of Physical Education, Shanghai University of Sport, Shanghai 200438, China; 4 School of Physical Education and Health, Gannan Normal University, Ganzhou 341003, China; 5 China Wushu School, Beijing Sport University, Beijing 100084, China; 6 Department of Physical Education, Fuzhou University, Fuzhou 350108, China

**Keywords:** chronic kidney disease, exercise, blood pressure, dose–response, network meta-analysis, exercise dose

## Abstract

Background: Uncertainty persists regarding the dose–response association between exercise exposure and blood pressure (BP) control in chronic kidney disease (CKD). This dose–response network meta-analysis examined whether different exercise modalities and model-estimated metabolic-equivalent exposure levels were associated with changes in systolic BP (SBP) and diastolic BP (DBP). Methods: Exercise dose was estimated as metabolic equivalents of task minutes per week (MET-min/week) from reported session duration, frequency, and intensity. Primary outcomes were changes in SBP and DBP, with exploratory subgroup analyses by dialysis status, baseline BP, and intervention duration. Results: The analysis included 26 randomized controlled trials (1218 participants). Aerobic exercise was associated with lower SBP, with the greatest model-estimated reduction at 880 MET-min/week (MD, −7.73 mm Hg). Resistance and mind–body exercise also showed model-estimated reductions, whereas combined aerobic-resistance training was not statistically significant. Dose–response estimates differed by dialysis status and intervention length, but several subgroup estimates were based on sparse data and wide uncertainty. Evidence certainty ranged from very low to high (CINeMA). Conclusions: Exercise interventions may be associated with BP reduction in CKD, but the identified MET-min/week ranges should be interpreted as exploratory, model-derived estimates rather than validated therapeutic thresholds. Better reported randomized trials are needed to confirm dose ranges, safety, adherence, and tolerability in dialysis and non-dialysis CKD populations.

## 1. Introduction

Chronic kidney disease (CKD) affects approximately 850 million people worldwide, representing a major global public health challenge [1]. Hypertension is present in 60–90% of CKD patients and serves as both a cause and consequence of kidney disease progression [2,3]. Elevated blood pressure in CKD patients is strongly associated with accelerated decline in kidney function, increased cardiovascular morbidity, and higher all-cause mortality [4,5]. For every 10 mm Hg increase in SBP above 120 mm Hg, CKD progression risk increases by approximately 17%, while cardiovascular event risk rises by 25% [6,7].

Notably, dialysis patients face an even greater burden, with 80–90% experiencing hypertension despite multi-drug regimens [3,8]. These findings underscore the importance of effective blood pressure management across all stages of CKD, from early-stage disease through end-stage renal disease requiring dialysis.

Antihypertensive medications remain the cornerstone of guideline-based blood pressure management in CKD [9], but their use is complicated by challenges unique to this population. Treatment-resistant hypertension is common, with 40–50% of CKD patients failing to achieve guideline-recommended blood pressure targets despite pharmacotherapy [10,11]. Commonly prescribed antihypertensive agents carry risks of adverse effects that are particularly concerning in CKD, including electrolyte disturbances (hyperkalemia with ACE inhibitors or ARBs), acute kidney injury, and hypotension-induced syncope [12,13]. Dialysis patients face additional complexity, with interdialytic hypertension from fluid retention and intradialytic hypotension from rapid fluid removal creating a narrow therapeutic window [8]. These limitations, combined with substantial pill burden (averaging 10–12 medications daily) [14], support the evaluation of safe, non-pharmacological adjunctive interventions.

Exercise is a cost-effective and widely accessible intervention with established cardiovascular and metabolic benefits [15,16]. Trials in CKD have shown improvements in cardiorespiratory fitness, muscle strength, and quality of life [16,17,18], but their effects on resting blood pressure remain less consistently characterized. Previous systematic reviews have largely focused on functional capacity and mortality [19], with limited attention to dose–response associations for SBP and DBP. Current KDIGO guidance encourages physical activity in people with CKD [20], but it does not provide BP-specific exercise dose thresholds or modality-specific guidance for dialysis and non-dialysis populations. Existing trials have reported inconsistent BP findings: one trial observed reductions in SBP and DBP after a 12-week exercise intervention [21], whereas another study of similar duration reported no significant changes [22]. This inconsistency may reflect differences in baseline BP, dialysis status, intervention modality, duration, supervision, adherence, and exercise intensity. Dialysis patients also present distinct challenges, including reduced exercise tolerance related to uremia, anemia, muscle wasting, fluid-status variation, and dialysis schedules [23,24]. These uncertainties support a dose–response synthesis, while also requiring cautious interpretation because available trials are clinically heterogeneous.

Therefore, we conducted a dose–response network meta-analysis to: (1) estimate associations between exercise dose and changes in SBP and DBP among patients with CKD; (2) compare model-estimated dose–response patterns across exercise modalities; and (3) explore whether dialysis status, baseline BP, and intervention duration modified these associations. Because the available evidence was expected to be heterogeneous and sparse in some subgroups, the resulting MET-min/week values were interpreted as exploratory, model-derived estimates rather than definitive treatment thresholds.

## 2. Results

### 2.1. Study Selection

**Figure 1:**
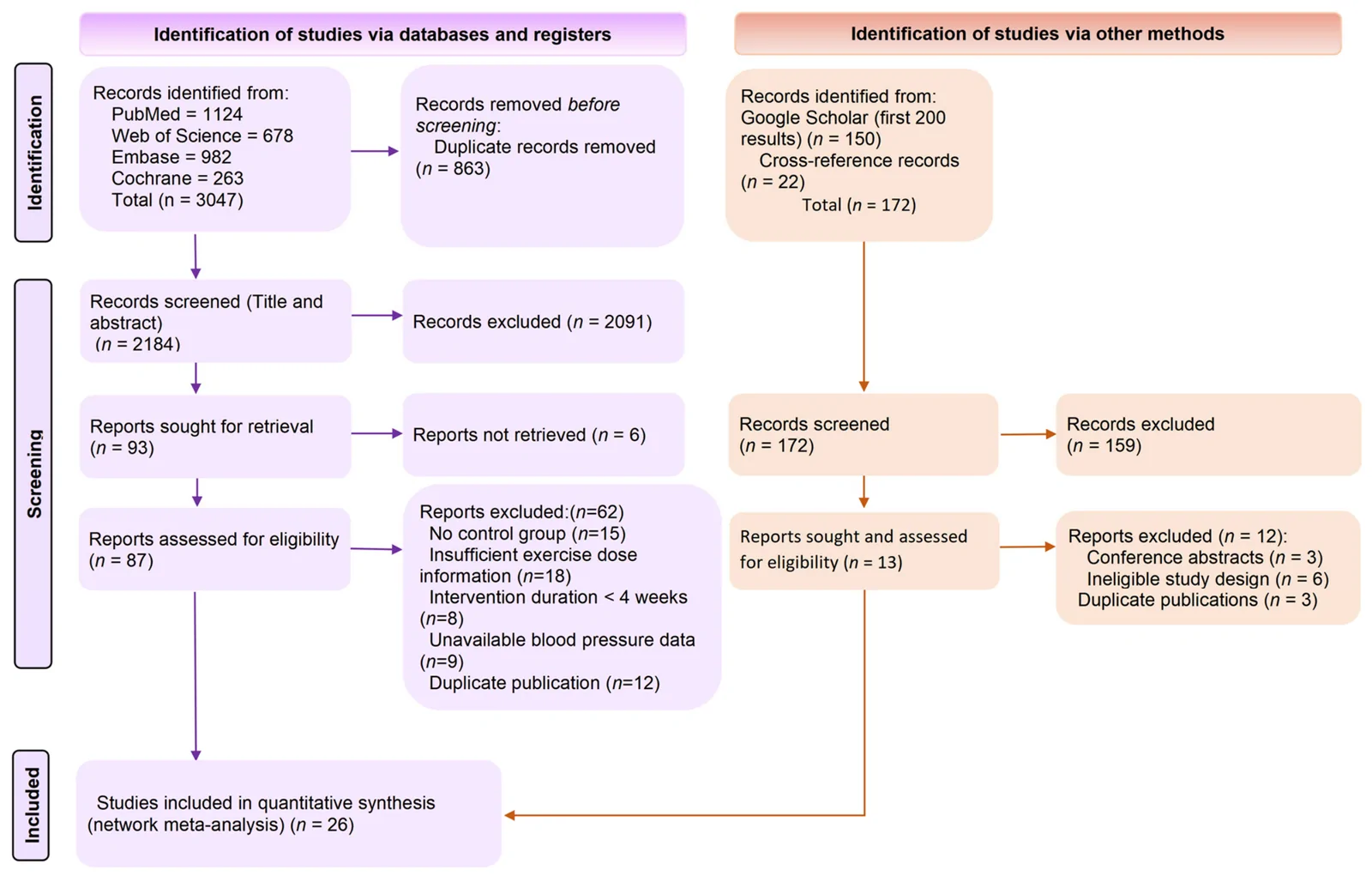
Screening and selection process of studies for the systematic review.

Two reviewers independently screened all retrieved records. A total of 3047 records were identified from four databases. After removing 863 duplicates, 2184 records underwent title and abstract screening, and 2091 were excluded as irrelevant. The remaining 93 reports were sought for retrieval. Six reports could not be retrieved, leaving 87 reports for full-text assessment. Of these, 62 reports were excluded because they had no control group (*n* = 15), insufficient exercise dose information (*n* = 18), intervention duration <4 weeks (*n* = 8), duplicate publication (*n* = 12), or unavailable blood pressure data (*n* = 9). Twenty-five studies met eligibility criteria, with one additional study identified from reference screening, resulting in 26 randomized controlled trials included in the final analysis. Disagreements were resolved by a third reviewer (Figure 1).

### 2.2. Study Characteristics

The 26 trials enrolled 1218 participants with chronic kidney disease, including 18 intervention arms (32.1%) in dialysis patients and 38 arms (67.9%) in non-dialysis patients. Participants were recruited from 12 countries, with a mean age of 55.95 years. Exercise interventions included aerobic exercise (15 arms), combined aerobic and resistance training (9 arms), resistance training (3 arms), and mind–body exercise (3 arms: yoga and tai chi). All control groups (25 arms) received usual care. Intervention length ranged from 12 to 52 weeks (median 24 weeks), with 2–7 sessions per week. Baseline mean systolic blood pressure was 140.3 mm Hg, with 91.1% of arms having baseline SBP ≥ 130 mm Hg. Detailed study characteristics, including reported CKD stage and dialysis status, are presented in Supplementary Table S1. Among studies reporting CKD stage, the included populations ranged from CKD stages 2 to 5.

### 2.3. Risk of Bias

We used the following criteria to assess the quality of studies. For the randomization process, although all RCTs involved random allocation, studies that did not report specific randomization methods (e.g., computer-generated) were rated as “some concerns.” Regarding deviations from the intended interventions, it is not possible to blind participants due to the nature of exercise studies. However, some studies reported blinding of allocation personnel and other staff, which helps reduce bias. Studies with no blinding of any person were rated as “high risk.” For studies with potential missing outcome data, they were rated as “some concerns” or “high risk.” For outcome measurement, all outcomes were assessed using objective measurement tools to measure blood pressure, so all studies were rated as “low risk.” For selective reporting, most studies were preregistered and reported according to predefined protocols. However, for studies that did not provide a registration number, this risk could not be assessed, and they were rated as “some concerns.” Finally, 2 studies were rated as “high risk of bias,” 13 studies as “some concerns,” and 11 studies as “low risk of bias.” Detailed risk of bias results are provided in Supplementary Section S4.

### 2.4. Hypothesis Testing and Model Selection

No evidence was found suggesting that the dose–response network meta-analysis violated the assumptions of connectivity (Supplementary Section S5.1), consistency (Supplementary Section S5.2), or transitivity (Supplementary Section S5.3). The quadratic function model was selected as the primary modeling approach based on model-fit criteria (Supplementary Section S5.4).

### 2.5. Dose–Response Relationship of Overall Exercise

**Figure 2:**
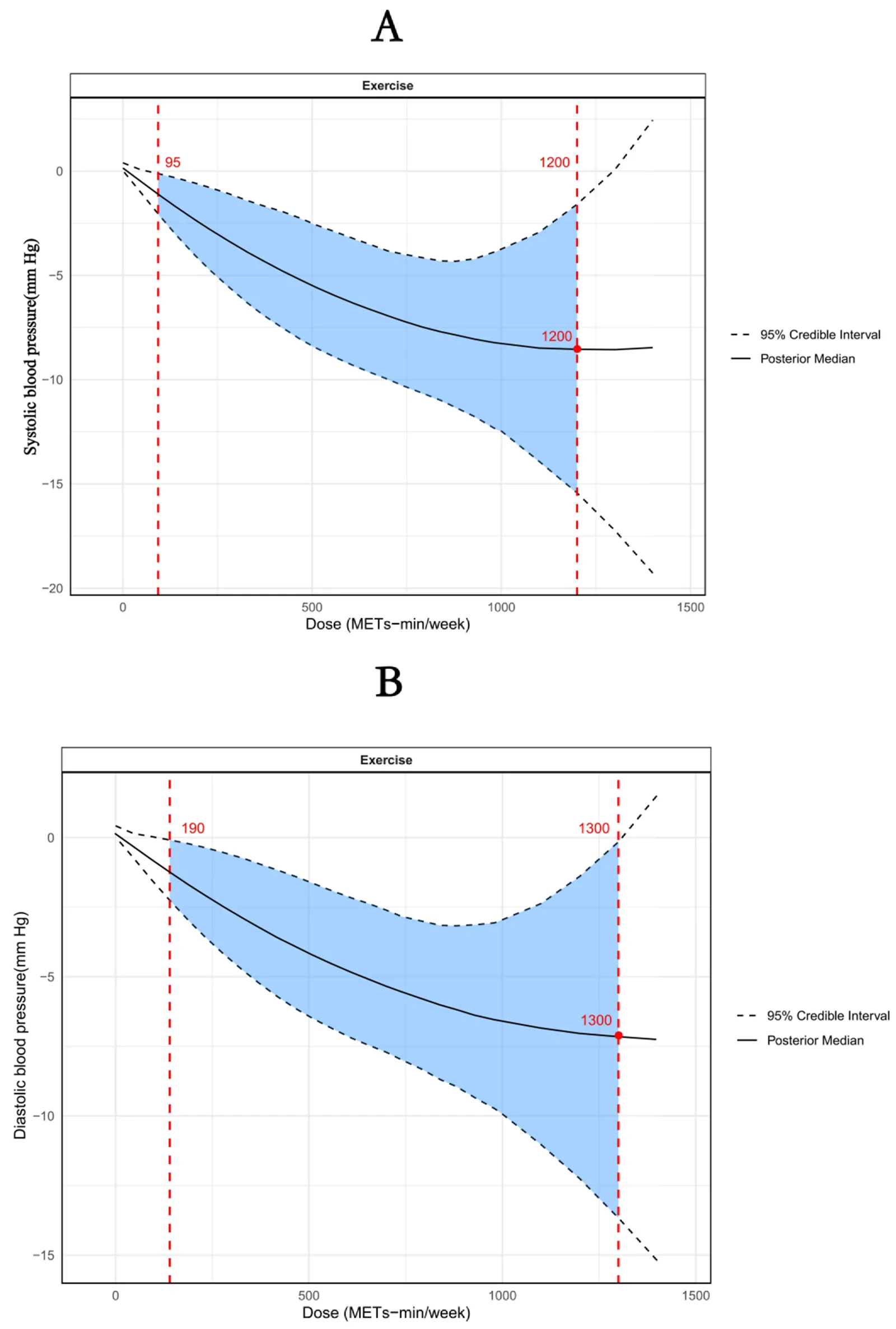
Overall dose–response relationships between exercise exposure and blood pressure in patients with CKD. (**A**) Systolic blood pressure; (**B**) Diastolic blood pressure.

Table 1 summarizes the dose–response network meta-analysis. The model suggested an association between greater exercise exposure and larger BP reductions across the tested dose ranges, although estimates near the lower and upper boundaries were supported by fewer observations. For SBP, statistically significant reductions began at 95 MET-min/week, with the greatest model-estimated reduction at 1200 MET-min/week (−8.53 mm Hg, 95% CrI: −15.43, −1.59), which was the upper limit of the tested range. For DBP, statistically significant effects began at 190 MET-min/week, with the greatest model-estimated reduction at 1300 MET-min/week (−7.30 mm Hg, 95% CrI: −14.17, −0.68). These values should therefore be interpreted as model-derived estimates within the available data rather than validated clinical thresholds. Figure 2 illustrates the overall dose–response relationships between exercise exposure and BP in CKD.

The red dashed lines represent the model-estimated lower and upper exposure values associated with statistically significant BP reductions, while the blue shaded area indicates the statistically significant region. Red dots represent the exposure value associated with the greatest predicted reduction within the modeled range. MET-min/week: metabolic equivalents of task minutes per week. Estimates near the extremes of the curve should be interpreted cautiously when supported by few observations.

**Figure 3:**
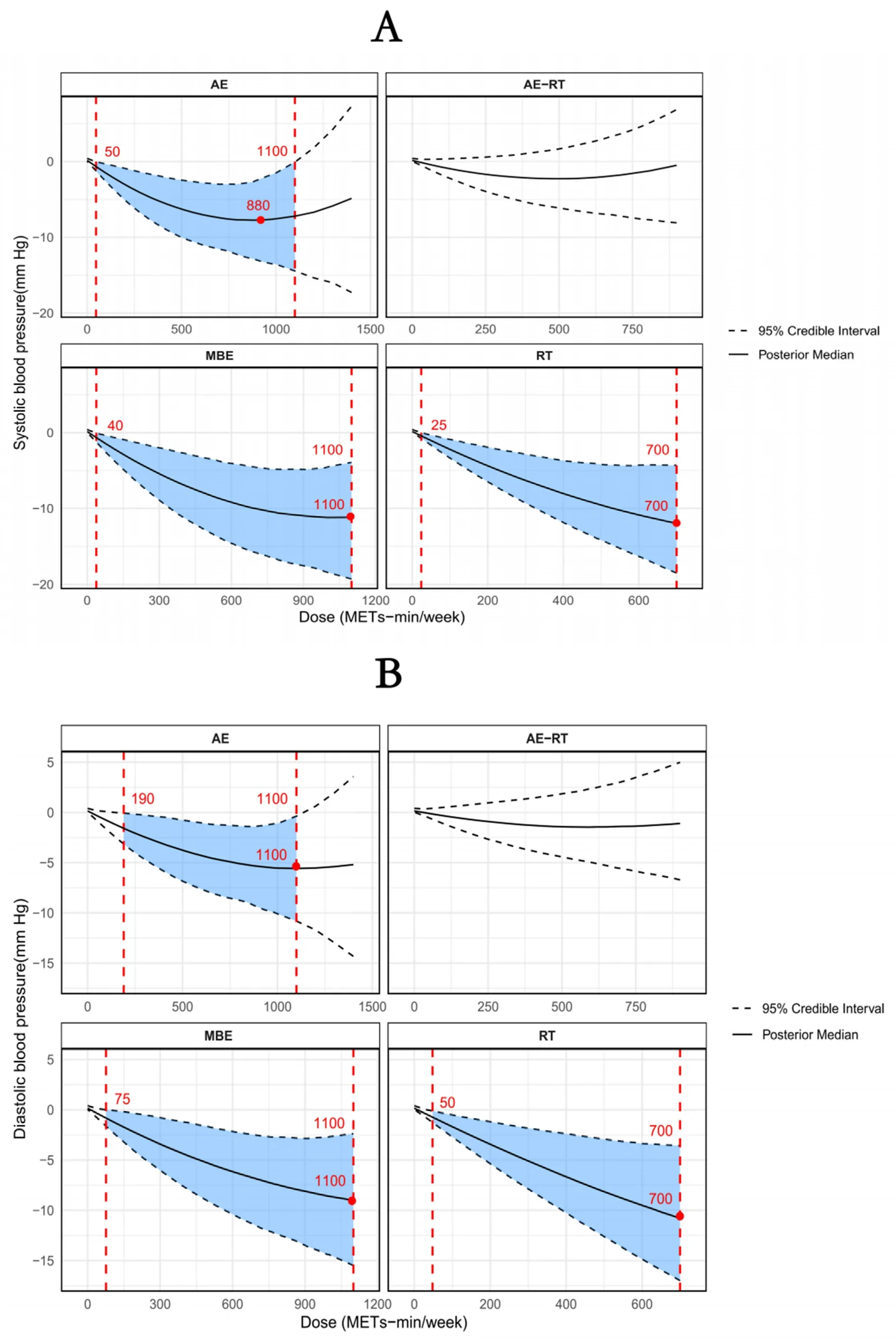
Dose–response relationships between exercise modalities and blood pressure in patients with CKD. (**A**) Systolic blood pressure; (**B**) Diastolic blood pressure.

The modality-specific dose–response curves are presented in Figure 3 and are summarized in the following section.

The red dashed lines represent the model-estimated lower and upper exposure values associated with statistically significant reductions, while the blue shaded area indicates the statistically significant region. Red dots indicate the exposure value associated with the greatest predicted reduction within the modeled range. MET-min/week: metabolic equivalents of task minutes per week. Estimates near the extremes of the curve should be interpreted cautiously when supported by few observations.

Exploratory analyses by dialysis status suggested different model-estimated exposure ranges between dialysis and non-dialysis populations. For SBP, dialysis studies showed statistically significant reductions across 560–1300 MET-min/week, with the greatest model-estimated reduction at 1300 MET-min/week (−13.73 mm Hg), whereas non-dialysis studies showed statistically significant reductions across 75–830 MET-min/week, with the greatest model-estimated reduction at 570 MET-min/week (−6.40 mm Hg). Similar patterns were observed for DBP (dialysis: 930–1400 MET-min/week; non-dialysis: 150–870 MET-min/week). Because these subgroup estimates were based on study-level data and heterogeneous CKD populations, they should not be interpreted as definitive dialysis-specific prescriptions.

Intervention duration also appeared to influence model-estimated outcomes, although interpretation is limited by uneven data distribution across duration categories. Interventions lasting >12 weeks were associated with statistically significant SBP reductions across 330–1200 MET-min/week, with the greatest model-estimated reduction at 1200 MET-min/week (−9.81 mm Hg). Interventions lasting ≤12 weeks showed statistically significant SBP reductions across 230–870 MET-min/week, with the greatest model-estimated reduction at 720 MET-min/week (−7.26 mm Hg). For DBP, shorter interventions showed larger model-estimated reductions than longer interventions; however, this finding should be interpreted cautiously because intervention design, intensity, and supervision varied across studies. Sensitivity analyses excluding high-risk studies showed broadly similar dose–response patterns (Supplementary Sections S5.5 and S5.6).

### 2.6. Dose–Response Relationship of Various Exercise Modalities

When stratified by exercise modality (Figure 3), RT, mind–body exercise, and AE were each associated with statistically significant BP reductions in the model, but the evidence base for several modality–dose combinations was sparse. For SBP, RT showed the greatest model-estimated reduction at 700 MET-min/week (−11.86 mm Hg, 95% CrI: −18.08, −4.31), which was the upper limit of its tested range. Mind–body exercise showed a comparable model-estimated reduction at 1000 MET-min/week (−11.31 mm Hg, 95% CrI: −19.28, −3.91), and AE showed statistically significant reductions across the broadest exposure range (45–1100 MET-min/week), with the greatest model-estimated reduction at 880 MET-min/week (−7.73 mm Hg, 95% CrI: −12.85, −2.67). For DBP, similar patterns were observed for RT (−10.68 mm Hg at 700 MET-min/week), mind–body exercise (−9.02 mm Hg at 1100 MET-min/week), and AE (−5.61 mm Hg at 1100 MET-min/week). AE-RT did not reach statistical significance for either SBP or DBP. This should not be interpreted as evidence that combined training is ineffective; rather, it may reflect heterogeneity in study design, baseline BP, intensity, duration, supervision, adherence, dialysis status, and the limited number of contributing studies.

### 2.7. Meta-Regression and Publication Bias

Meta-regression did not reveal that age, baseline blood pressure or intervention length significantly moderated the effects of exercise on SBP and DBP (Supplementary Section S5.8).

Supplementary Section S6 presents a detailed assessment of publication bias for SBP and DBP. The funnel plots appeared generally symmetrical, and Begg’s tests indicated no statistically significant asymmetry (*p* > 0.05), suggesting no clear evidence of small-study effects.

### 2.8. Certainty of Evidence

CINeMA assessments indicated that the evidence for the impact of various exercise types on SBP and DBP was rated as very low to high (Supplementary Section S7), with the main reasons for downgrading including within-study bias, imprecision, incoherence, and heterogeneity.

### 2.9. Model-Derived Exercise Accumulation Estimates

Table 2 presents model-derived weekly exercise accumulation estimates for six exercise modes. To enhance interpretability, activity-specific MET values from the Adult Compendium of Physical Activities were used to convert MET-min/week values into approximate minutes per week. These estimates should be viewed as illustrative conversions, not as validated prescriptions. From the available model and CINeMA assessments, RT and mind–body exercise ranked favorably, but certainty varied across comparisons. Yoga, tai chi, and RT had higher certainty ratings in the available network than several aerobic modalities, whereas cycling, jogging, and walking showed very low certainty. These differences should be interpreted in light of sparse modality-specific evidence and possible exposure misclassification.

Dialysis status remains clinically important, but the estimated higher exposure ranges in dialysis populations should be interpreted cautiously. Non-dialysis studies showed lower model-estimated exposure ranges than dialysis studies, but the two groups differed in CKD stage, functional capacity, intervention setting, and likely comorbidity burden. Any exercise plan should therefore be individualized according to functional capacity, dialysis schedule, comorbidities, medication use, BP stability, and safety monitoring rather than based solely on the model-derived MET-min/week values.

## 3. Discussion

### 3.1. Principal Findings and Implications

This analysis suggests that RT, mind–body exercise (MBE), and AE may be associated with reductions in SBP among patients with CKD. The dose–response model indicated larger predicted reductions at higher exposure levels within the tested range; however, several estimates occurred near the upper boundary of the modeled exposure range and should be interpreted cautiously. RT, MBE, and AE ranked favorably in modality-specific analyses, whereas AE-RT did not reach statistical significance. The absence of a statistically significant effect for AE-RT may reflect sparse evidence and heterogeneity rather than a true lack of benefit. Dialysis status and baseline BP appeared to influence the model-estimated exposure-response patterns, but these findings are exploratory and based on study-level rather than participant-level data.

For DBP, the direction of findings was broadly consistent with the SBP analysis. RT, MBE, and AE were associated with model-estimated DBP reductions, whereas AE-RT did not reach statistical significance. Participants with higher baseline DBP appeared more likely to show statistically significant reductions, while those with baseline DBP < 80 mm Hg showed limited evidence of benefit. Shorter interventions showed larger model-estimated DBP reductions than longer interventions, but this comparison is vulnerable to differences in trial design, supervision, intensity progression, and sample size. These findings should therefore be considered hypothesis-generating.

### 3.2. Interpretation and Comparison with Existing Literature

These findings are broadly consistent with previous systematic reviews showing that exercise can improve functional and cardiovascular outcomes in CKD, while BP-specific effects have remained uncertain. Heiwe and Jacobson reported benefits for cardiorespiratory fitness and health-related quality of life [25], whereas more recent syntheses in dialysis populations have noted low-certainty evidence for BP outcomes because of small samples and heterogeneous interventions [26]. By modeling exercise exposure as MET-min/week, the present review provides an exploratory quantitative description of dose–response patterns. This approach may help explain some heterogeneity across trials, but it does not establish validated BP-lowering thresholds for CKD or dialysis populations.

The magnitude of the model-estimated BP reductions may be clinically relevant if replicated in adequately powered trials. However, direct comparisons with antihypertensive medications or projections of cardiovascular events prevented are indirect and were therefore not used as evidence for clinical efficacy in the revised interpretation. Exercise should be considered a potential adjunct to guideline-based BP management rather than a substitute for pharmacological treatment or individualized nephrology care. Future trials should determine whether exercise-induced BP changes translate into improvements in cardiovascular outcomes, kidney function decline, hospitalization, or mortality.

RT and mind–body exercises ranked favorably in the model, but the available evidence does not justify concluding that they are clinically superior to AE or AE-RT. Their apparent advantages may reflect differences in participant characteristics, baseline BP, supervision, adherence, intervention duration, or the limited number of trials in each modality. The non-significant findings for AE-RT should also be interpreted cautiously, because combined training protocols varied substantially and may have included lower effective intensity, different progression schemes, or populations with lower baseline BP. Current KDIGO guidance supports physical activity in CKD [20], and the present analysis may inform future research on modality-specific exercise design rather than replace guideline recommendations.

From a mechanistic perspective, the favorable model-estimated association for resistance training may be biologically plausible in CKD, but it should not be interpreted as proof of superiority. Protein-energy wasting and loss of muscle mass are common in CKD, with reported prevalence increasing in dialysis populations [27]. Resistance training may help counter muscle weakness and metabolic dysfunction through effects on muscle mass, insulin sensitivity, inflammation, and vascular function [18,28,29]. However, the current analysis cannot determine which mechanisms explain the observed BP changes, and future trials should measure body composition, inflammatory markers, vascular function, adherence, and adverse events alongside BP outcomes.

The dialysis subgroup findings require particular caution. Higher model-estimated exposure ranges in dialysis studies may reflect the underlying severity and physiology of dialysis populations, including volume overload, interdialytic fluid shifts, autonomic dysfunction, anemia, muscle wasting, uremic toxin accumulation, and hemodynamic instability during or after dialysis [3,8,23,30]. At the same time, these factors may reduce exercise tolerance and increase the need for individualized safety assessment. Therefore, the higher estimated MET-min/week values in dialysis patients should not be translated directly into high-intensity exercise recommendations without careful monitoring of symptoms, BP stability, adherence, and adverse events.

For clinical interpretation, Table 2 converts selected model-derived MET-min/week values into approximate weekly exercise accumulation. These conversions are intended to make the exposure scale understandable, not to prescribe fixed regimens. For example, the estimates for RT, tai chi, yoga, walking, cycling, or jogging depend on assumed compendium MET values and may not reflect relative intensity in older, frail, anemic, sarcopenic, or dialysis-dependent patients. Exercise plans in CKD should be individualized according to functional capacity, comorbidities, medication use, dialysis timing, BP stability, and patient preference, and should be adapted gradually with safety monitoring.

Finally, while this analysis focused on resting BP, an important knowledge gap remains regarding exercise-induced BP responses during exertion. Recent evidence suggests that SBP responses relative to work rate during incremental exercise may predict future hypertension risk in general populations [31]. Whether similar relationships exist in CKD, and whether training modifies exertional BP responses, remains uncertain. Longer and better reported trials are needed to assess cardiovascular outcomes, kidney function progression, safety, adherence, and mortality.

### 3.3. Limitations and Strengths

This study has several limitations. First, the number of included studies was modest relative to the complexity of the analyses, and several subgroup and modality-specific estimates were based on sparse data. Estimates at very low (<100 MET-min/week) and very high (>1200 MET-min/week) exposure levels are therefore exploratory. Second, although CKD stage and dialysis status were extracted when reported, the primary studies did not consistently provide stage-specific participant counts, CKD etiology, kidney function indices (e.g., eGFR and albuminuria), dialysis modality or vintage, or detailed comorbidity profiles. Reporting was also incomplete for BMI, waist-to-hip ratio, diabetes status, cardiovascular risk, antihypertensive medication use, BP measurement protocols, adherence, dropouts, and adverse events. These reporting limitations prevented a more granular clinical characterization of the pooled population and more detailed adjustment for clinical heterogeneity. Third, MET-min/week values were derived from the Adult Compendium of Physical Activities and therefore represent absolute, population-based estimates. They may misclassify relative exercise intensity in CKD patients who are older, frail, sarcopenic, anemic, or dialysis-dependent. Broad exercise categories such as tai chi, yoga, resistance training, and combined training may also include substantial within-category variation. Fourth, although searches were run without database language filters, reliable extraction required English full text or sufficient English-language data, introducing potential language bias. Fifth, exercise accumulation estimates derived from pooled data reflect population-level model estimates and should not be directly generalized to individual patients without clinical assessment.

Despite these limitations, the study has several strengths. It used a prespecified dose–response network meta-analytic framework to synthesize randomized evidence in CKD, considered dialysis status and baseline BP as clinically relevant study-level modifiers, and assessed confidence in the network estimates using CINeMA. The analysis provides a structured description of model-estimated exposure-response patterns that can guide future trial design and reporting.

## 4. Materials and Methods

### 4.1. Protocol and Registration

This study represents a prospectively registered network meta-analysis and systematic review (PROSPERO registration number: CRD420261295745), reported in accordance with the PRISMA 2020 statement and the PRISMA extension for network meta-analyses [32,33].The completed PRISMA 2020 checklist is provided as Supplementary File S2.

### 4.2. Search Strategy

Electronic databases searched for the literature review included PubMed, Web of Science, Embase, and the Cochrane Central Register of Controlled Trials, covering the period from inception to 28 January 2026. Searches were run without database language filters. The search strategy used relevant keywords and MeSH terms related to participants (chronic kidney disease), intervention (exercise), outcome (blood pressure), and study design (randomized controlled trials), combined using the logical operator AND. During full-text assessment, studies were included when an English full text or sufficient English-language data were available for reliable extraction; the potential language bias introduced by this requirement is acknowledged in the Limitations. A manual search of reference lists from relevant studies was also conducted. Detailed search strategies for each database are provided in Supplementary Section S1.

### 4.3. Inclusion and Exclusion Criteria

Eligibility criteria were established using the PICOS format (population, intervention, comparison, outcomes, study design) and reported in accordance with PRISMA 2020 and PRISMA-NMA [32,33].

The inclusion criteria were as follows: (1) randomized controlled trials (including crossover trials with first-period data) evaluating structured exercise interventions; (2) studies enrolling adults (≥18 years) diagnosed with chronic kidney disease (CKD, stages 1–5, including dialysis patients), defined according to contemporary KDIGO criteria (e.g., eGFR < 60 mL/min/1.73 m^2^ for ≥3 months and/or evidence of kidney damage) [20]; (3) studies involving any form of exercise training (e.g., aerobic, resistance, combined, or mind–body exercise), conducted either during or outside dialysis sessions, with sufficient reporting of exercise dose (frequency, duration, intensity) to allow calculation of weekly MET-min; and 4) studies reporting resting systolic and/or diastolic blood pressure measured at baseline and post-intervention. Although adults with CKD stages 1–5 were eligible for inclusion, none of the included studies explicitly reported enrolling participants with stage 1 CKD.

The exclusion criteria were as follows: studies involving acute kidney injury, recent kidney transplant recipients (<6 months), or terminal illness; studies combining exercise with other complex interventions (e.g., diet or medication changes) or assessing only acute exercise effects (<4 weeks); studies without a control/comparison group; studies reporting only categorical blood pressure outcomes without continuous data; non-randomized designs (e.g., cohort or before-after studies); duplicates, conference abstracts, reviews, commentaries, or other non-original research; and non-English full-text publications for which reliable data extraction was not feasible.

### 4.4. Data Extraction and Coding

Two reviewers independently extracted data using a predesigned Excel form. Extracted items included study characteristics (author, year, region), participant characteristics (age, sample size, CKD stage, dialysis status, and available clinical characteristics), intervention details (exercise type, frequency, session duration, intensity, supervision, and intervention length), control conditions, BP outcomes, adherence, dropouts, and adverse events when reported. Mean differences (MDs) and standard deviations (SDs) for changes from baseline to post-intervention were obtained for both exercise and control groups. SDs for change scores were estimated using a correlation coefficient of 0.5, consistent with typical measurement reliability [34]. Missing SDs were derived from confidence intervals, *p* values, or t values; when unavailable, corresponding authors were contacted. For dose–response analyses, standard errors (SEs) were required and calculated from SDs using the following formula:

$$\mathrm{SE}\;=\;\frac{\mathrm{SD}}{\sqrt{N}}$$

where N denotes sample size. Data accuracy was independently cross-checked, and the final dataset was verified by a third reviewer.

For the dose–response network meta-analysis, interventions were categorized at three levels. At the first level, interventions were classified as exercise or control. At the second level, exercise interventions were categorized by modality as reported in the original studies: aerobic exercise (AE), resistance training (RT), combined aerobic and resistance training (AE-RT), and mind–body exercise. At the third level, interventions were classified by total weekly exercise exposure, expressed as metabolic equivalents of task minutes per week (MET-min/week). For duration, warm-up and cool-down periods were excluded when separable from the active exercise period. If exercise duration progressed over time, the average active session duration was used. MET values were assigned according to the reported modality, intensity, and session characteristics using the 2024 Adult Compendium of Physical Activities [35]. When original reports did not provide enough detail to identify an exact activity code, the closest conservative compendium category was selected and recorded. For broad modalities such as tai chi, yoga, resistance training, and combined training, the assigned MET value was based on the reported intensity, supervision, and exercise description where available. The allocation process and arm-level dose information are provided in Supplementary Section S2.

Based on estimated MET-min/week, each intervention was assigned to the nearest predefined dose category to maintain network connectivity and ensure a sufficient number of observations per dose node. Exact calculated doses were retained in the extraction dataset and are shown in the Supplementary Material. This categorization was used for model stability and should be interpreted as an approximation of exercise exposure rather than a patient-specific measure of relative exercise intensity.

### 4.5. Statistical Analysis

Dose–response relationships between exercise dose and exercise modality were analyzed using the MBNMAdose package in R (version 4.5.2), with curves visualized using ggplot2. Because SBP and DBP are continuous outcomes measured in the same unit (mm Hg), mean differences (MDs) were used to quantify change between intervention and control groups. Before conducting the dose–response network meta-analysis, key assumptions were evaluated, including network connectivity, consistency, and transitivity [36,37,38]. Multiple functional forms were fitted to characterize dose–response associations, including Emax, restricted cubic spline, log-linear, non-parametric, exponential, quadratic, spline, and fractional polynomial models. Model performance was assessed using the deviance information criterion (DIC), residual standard deviation, model complexity, and residual deviance. The model with the lowest DIC and acceptable residual fit was selected as the primary model, consistent with model-based and dose–response network meta-analysis methods [39,40]. Given expected between-study heterogeneity, random-effects models were applied. Bayesian model outputs are reported as posterior median MDs with 95% credible intervals (CrIs). Effects were considered statistically significant when the 95% CrI excluded zero. The exposure value associated with the greatest predicted BP reduction within the modeled range was reported as a model-derived estimate, not as a validated therapeutic threshold. For multi-arm trials with more than one eligible exercise arm sharing a common comparator, all eligible arms were retained within the network model while accounting for the shared study identifier so that the common comparator group was not treated as independent multiple times. In supplementary pairwise displays or tabulations, shared comparators were handled to avoid duplicate counting of the same participants.

### 4.6. Additional Analyses

Sensitivity analyses excluding studies with high risk of bias or those including educational components were performed to assess the robustness of the results. Subgroup analyses were conducted to explore potential effect modification by baseline BP, using standard thresholds (SBP ≥ 140 vs. <140 mm Hg; DBP ≥ 80 vs. <80 mm Hg) [41]. Recognizing the distinct pathophysiology and reduced exercise capacity in dialysis populations, we performed prespecified subgroup analyses stratified by dialysis status. Dialysis patients were defined as those receiving maintenance hemodialysis or peritoneal dialysis, while non-dialysis patients were defined as CKD patients not requiring renal replacement therapy. We also conducted subgroup analyses based on intervention length (≤12 vs. >12 weeks), because 12-week training periods are common in CKD exercise studies and several included trials clustered around this duration [42]. These subgroup analyses were study-level and exploratory because participant-level data on CKD etiology, BMI, diabetes status, comorbidities, medication use, and cardiovascular risk were not consistently available.

Network meta-regressions were performed to examine whether selected study-level covariates modified the intervention effects [43]. Covariates of interest included average age, intervention length, and baseline BP. Posterior median regression coefficients with 95% CrIs were estimated, with CrIs crossing zero indicating no statistically significant evidence of effect modification. Network meta-regressions were conducted using the R package MBNMAdose. Publication bias and small-study effects were assessed using funnel plots and Begg’s test, with symmetrical plots and *p* values ≥ 0.05 interpreted as showing no clear evidence of small-study effects [44].

### 4.7. Risk of Bias and Certainty of Evidence

Risk of bias was evaluated using the Cochrane Risk of Bias tool 2.0 (RoB 2), which covers five domains: randomization, deviations from intended interventions, missing outcome data, outcome measurement, and selective reporting [45]. Studies were classified as low risk, some concerns, or high risk. Two reviewers independently performed the assessment, with disagreements resolved through discussion or consultation with a third reviewer.

Confidence in the results was assessed using the Confidence in Network Meta-Analysis (CINeMA) web application, which rates each comparison based on within-study bias, reporting bias, indirectness, imprecision, heterogeneity, and inconsistency, following recommended guidelines. Evidence was graded as high, moderate, low, or very low [46].

## 5. Conclusions

This systematic review and dose–response network meta-analysis suggests that exercise interventions may be associated with reductions in SBP and DBP among patients with CKD. However, the model-estimated dose–response patterns within the observed dose range should be interpreted cautiously because sparse evidence and clinical and methodological heterogeneity, particularly differences between dialysis and non-dialysis populations, limit comparability and generalizability. The identified MET-min/week values should therefore be regarded as provisional model-based anchors for future research rather than validated treatment thresholds or individualized exercise prescriptions. Further well-reported randomized trials are needed to clarify safety, adherence, tolerability, and applicability across CKD populations.

## Figures and Tables

**Table 1:** Summary of dose–response network meta-analysis results.

Category	Data Point	Blood Pressure	Model-Estimated Statistically Significant Range (MET-min/Week)	Exposure Associated with Greatest Model-Estimated Reduction (MET-min/Week)	Posterior Median MD and 95% CrI at That Exposure
Overall exercise	56	SBP	95–1200	1200	−8.53 mm Hg (−15.43, −1.59)
	54	DBP	190–1300	1300	−7.30 mm Hg (−14.17, −0.68)
Baseline SBP (≥140 mmHg)	23	SBP	230–1200	1100	−8.70 mm Hg (−15.77, −2.27)
Baseline SBP (<140 mmHg)	19	SBP	60–530	370	−7.43 mm Hg (−12.75, −0.90)
Baseline DBP (≥80 mmHg)	33	DBP	95–1300	1100	−7.77 mm Hg (−12.03, −3.12)
Baseline DBP (<80 mmHg)	10	DBP	NA	NA	NA
Dialysis patients	18	SBP	560–1300	1300	−13.73 mm Hg (−25.97, −1.18)
Non-dialysis patients	38	SBP	75–830	570	−6.40 mm Hg (−9.73, −2.63)
Dialysis patients	18	DBP	930–1400	1400	−8.80 mm Hg (−15.72, −1.65)
Non-dialysis patients	36	DBP	150–870	760	−5.57 mm Hg (−8.98, −1.82)
Length (>12 weeks)	40	SBP	330–1200	1200	−9.81 mm Hg (−18.77, −0.65)
Length (≤12 weeks)	16	SBP	230–870	720	−7.26 mm Hg (−13.17, −1.65)
Length (>12 weeks)	38	DBP	610–1000	1000	−5.04 mm Hg (−9.28, −0.45)
Length (≤12 weeks)	16	DBP	420–1100	1100	−9.74 mm Hg (−16.74, −2.58)
Sensitivity analysis	51	SBP	50–1200	1100	−8.73 mm Hg (−14.51, −3.01)
	49	DBP	140–1300	1300	−7.14 mm Hg (−13.67, −0.16)
AE	23	SBP	45–1100	880	−7.73 mm Hg (−12.85, −2.67)
	20	DBP	190–1100	1100	−5.61 mm Hg (−10.81, −0.37)
AE-RT	20	SBP	NA	NA	NA
	18	DBP	NA	NA	NA
Mind–body exercise	28	SBP	40–1100	1000	−11.31 mm Hg (−19.28, −3.91)
	27	DBP	75–1100	1100	−9.02 mm Hg (−15.48, −2.37)
RT	29	SBP	25–700	700	−11.86 mm Hg (−18.08, −4.31)
	28	DBP	50–700	700	−10.68 mm Hg (−16.98, −3.58)

NA: not applicable (no statistically significant effect observed); AE: aerobic exercise; AE-RT: combined aerobic and resistance training; RT: resistance training; MET-min/week: metabolic equivalents of task minutes per week; SBP: systolic blood pressure; DBP: diastolic blood pressure; CrI: credible interval. Exploratory subgroup analyses suggested that baseline BP may influence model-estimated dose–response patterns. Among participants with baseline SBP ≥ 140 mm Hg, statistically significant reductions were observed across 230–1200 MET-min/week, with the greatest model-estimated reduction of −8.70 mm Hg (95% CrI: −15.77, −2.27) at 1100 MET-min/week. Among those with SBP < 140 mm Hg, statistically significant reductions were observed across 60–530 MET-min/week, with the greatest model-estimated reduction of −7.43 mm Hg at 370 MET-min/week. For DBP, statistically significant reductions were observed only among participants with baseline DBP ≥ 80 mm Hg (95–1300 MET-min/week; greatest model-estimated reduction, −7.77 mm Hg at 1100 MET-min/week), whereas no statistically significant relationship was observed in those with DBP < 80 mm Hg.

**Table 2:** Model-derived exercise accumulation estimates for BP reduction.

Outcome	Exercise Mode	Assigned MET Value (Compendium Code)	Model-Derived Range (min/Week)	Minutes/Week at Greatest Model-Estimated Reduction	CINeMA Certainty
SBP	Cycling	7.0 (01016)b	5–155	125	Very low
	Jogging	4.8 (12025)c	10–230	185	Very low
	Walking	3.5 (17160)d	15–315	250	Very low
	Resistance training	3.5 (02054)e	5–200	200	Moderate
	Yoga	2.3 (02175)	15–480	435	High
	Tai chi	3.3 (15670)	10–335	305	High
DBP	Cycling	7.0 (01016)b	25–155	155	Very low
	Jogging	4.8 (12025)c	40–230	230	Very low
	Walking	3.5 (17160)d	55–315	315	Very low
	Resistance training	3.5 (02054)e	15–200	200	Moderate
	Yoga	2.3 (02175)	30–480	480	High
	Tai chi	3.3 (15670)	20–335	335	High

Note: a: Assigned MET values were coded using the 2024 Adult Compendium of Physical Activities. b: Cycling: self-selected moderate pace. c: Jogging, in place. d: Walking for pleasure. e: Resistance (weight) training, multiple exercises, 8–15 repetitions at varied resistance. Values of 3–6 METs represent moderate-intensity activity and >6 METs represent vigorous-intensity activity in absolute terms. The converted minutes/week are illustrative model-derived estimates and should not be interpreted as individualized exercise prescriptions. SBP: systolic blood pressure; DBP: diastolic blood pressure; MET: metabolic equivalent of task; CINeMA: Confidence in Network Meta-Analysis.

## Data Availability

Data available on request from the authors. The data that support the findings of this study are available from the corresponding author, J.W., upon reasonable request.

## Supplementary Materials

The following supporting information can be downloaded at: https://www.mdpi.com/article/10.3390/life16081243/s1, Supplementary File S1: Supplementary methods and results; Figure S1: The detail of risk of bias; Figure S2: The summary of risk of bias; Figure S3: Dose-level network (systolic blood pressure); Figure S4: Dose-level network (diastolic blood pressure); Figure S5: Agent-level network (systolic blood pressure); Figure S6: Agent-level network (diastolic blood pressure); Figure S7: “Split” network meta-analysis of different exercise interventions on systolic blood pressure; Figure S8: Dose–response relationship between different exercise (METs-min/week) and systolic blood pressure (quadratic function); Figure S9: Dose–response relationship between exercise and systolic blood pressure (≥140 mm Hg) at baseline; Figure S10: Dose–response relationship between exercise and systolic blood pressure (<140 mm Hg) at baseline; Figure S11: Dose–response relationship between exercise and systolic blood pressure (non-dialysis population); Figure S12: Dose–response relationship between exercise and systolic blood pressure (dialysis population); Figure S13: Dose–response relationship between exercise and systolic blood pressure (intervention length > 12 weeks); Figure S14: Dose–response relationship between exercise and systolic blood pressure (intervention length ≤ 12 weeks); Figure S15: Dose–response relationship between exercise and diastolic blood pressure (baseline blood pressure ≥ 80 mmHg); Figure S16: Dose–response relationship between exercise and diastolic blood pressure (baseline blood pressure < 80 mmHg); Figure S17: Dose–response relationship between exercise and diastolic blood pressure (non-dialysis population); Figure S18: Dose–response relationship between exercise and diastolic blood pressure (dialysis population); Figure S19: Dose–response relationship between exercise and diastolic blood pressure (intervention length > 12 weeks); Figure S20: Dose–response relationship between exercise and diastolic blood pressure (intervention length ≤ 12 weeks); Figure S21: Dose-response relationship between exercise and systolic blood pressure in patients with CKD after excluding high-risk studies and studies with education components; Figure S22: Dose-response relationship between exercise and diastolic blood pressure in patients with CKD after excluding high-risk studies and studies with education components; Figure S23: Funnel plot (systolic blood pressure).; Figure S24: Funnel plot (diastolic blood pressure); Figure S25: Network plot of study limitations of the included studies (systolic blood pressure); Figure S26: Network plot of study limitations of the included studies (diastolic blood pressure); Table S1: Study characteristics; Table S2: Systolic blood pressure Comparison of consistency and UME model fit; Table S3: Diastolic blood pressure Comparison of consistency and UME model fit; Table S4: Systolic blood pressure Node-splitting analysis of consistency; Table S5: Diastolic blood pressure Node-splitting analysis of consistency; Table S6: Systolic blood pressure Models fit comparison; Table S7: Diastolic blood pressure Models fit comparison; Table S8: Systolic blood pressure; Table S9: Diastolic blood pressure; Table S10: Dose-response network meta-regression (systolic blood pressure); Table S11: Dose-response network meta-regression (diastolic blood pressure); Table S12: The confidence of evidence using the CINeMA (systolic blood pressure); Table S13: The confidence of evidence using the CINeMA (diastolic blood pressure); Table S14: Summary of dose-response network meta-analysis result; Table S15: Model-derived exercise accumulation estimates for blood pressure reduction; Supplementary File S2: Completed PRISMA 2020 checklist. The studies summarized in Supplementary Table S1 are cited in the main reference list [22,47,48,49,50,51,52,53,54,55,56,57,58,59,60,61,62,63,64,65,66,67,68,69,70,71].
