# Supplementary material for: Dose–Response Effects of Different Exercise Modalities on Blood Pressure Control in Patients with Chronic Kidney Disease: A Systematic Review and Network Meta-Analysis
Source: Life (Basel). 2026 Jul 27;16(8):1243. doi: 10.3390/life16081243 (PMC13514883; doi:10.3390/life16081243)
Supplement: Supplementary file 1 [file life-16-01243-s001.zip › Supplementary File S1.pdf]

Supplementary Content

1. SEARCH STRATEGY 3

1.1 PUBMED 3
1.2 WEB OF SCIENCE 3
1.3 EMBASE 4
1.4 COCHRANE 5

2. ENCODING EXAMPLE 7

3. TABLE S1. STUDY CHARACTERISTICS 9

4. RISK OF BIAS 12

5. DOSE-RESPONSE NETWORK META-ANALYSES 13

5.1 Connectivity 13
5.2 Consistency 15
5.3 Transitivity 16
5.4 Model selection (systolic blood pressure as an example) 20
5.5 Subgroup analysis 24
5.5.1 Systolic blood pressure (baseline blood pressure ≥140 mmHg) 24
5.5.2 Systolic blood pressure (baseline blood pressure <140 mmHg) 24
5.5.3 Systolic blood pressure (non-dialysis population) 25
5.5.4 Systolic blood pressure (dialysis population) 26
5.5.5 Systolic blood pressure (intervention length ＞12 weeks) 27
5.5.6 Systolic blood pressure (intervention length ≤12 weeks) 28
5.5.7 Diastolic blood pressure (baseline blood pressure ≥80 mmHg) 29
5.5.8 Diastolic blood pressure (baseline blood pressure <80 mmHg) 30
5.5.9 Diastolic blood pressure (non-dialysis population) 31
5.5.10 Diastolic blood pressure (dialysis population) 32
5.5.11 Diastolic blood pressure (intervention length ＞12 weeks) 32
5.5.12 Diastolic blood pressure (intervention length ≤12 weeks) 33
5.6 SENSITIVITY ANALYSIS 34
5.6.1 Systolic blood pressure 34
5.6.2 Diastolic blood pressure 35
5.7 RANKING OF MODEL-ESTIMATED EXERCISE MODALITIES AND DOSES 36
5.7.1 Table S8. Systolic blood pressure 36
5.7.2 Table S9. Diastolic blood pressure 38
5.8.2 Diastolic blood pressure 42

6. PUBLICATION BIAS 42

6.1 SYSTOLIC BLOOD PRESSURE 42
6.2 DIASTOLIC BLOOD PRESSURE 43

7. GRADE SUMMARY OF ALL STUDIES 44

7.1 WITHIN-STUDY 44
7.2 REPORTING BIAS 44
7.3 INDIRECTNESS 45
7.4 IMPRECISION 45
7.5 HETEROGENEITY 45
7.6 INCOHERENCE 45
7.7 SUMMARIZING JUDGMENTS ACROSS CINeMA DOMAINS 45

1. Search strategy

1.1 Pubmed

("Renal Insufficiency, Chronic"[Mesh] OR "Kidney Diseases"[Mesh] OR chronic kidney disease[Title/Abstract] OR CKD[Title/Abstract] OR chronic renal disease[Title/Abstract] OR chronic renal insufficiency[Title/Abstract] OR chronic kidney failure[Title/Abstract] OR renal impairment[Title/Abstract] OR diabetic nephropathy[Title/Abstract] OR renal disease[Title/Abstract] OR end-stage renal disease[Title/Abstract] OR ESRD[Title/Abstract] OR hemodialysis[Title/Abstract] OR haemodialysis[Title/Abstract] OR peritoneal dialysis[Title/Abstract])

AND

("Exercise"[Mesh] OR "Exercise Therapy"[Mesh] OR "Motor Activity"[Mesh] OR "Sports"[Mesh] OR "Resistance Training"[Mesh] OR "Yoga"[Mesh] OR "Tai Ji"[Mesh] OR "Walking"[Mesh] OR "Physical Exertion"[Mesh] OR physical activity[Title/Abstract] OR movement[Title/Abstract] OR fitness[Title/Abstract] OR aerobic exercise[Title/Abstract]OR "high-intensity interval training"[Title/Abstract] OR "HIIT"[Title/Abstract]) OR resistance training[Title/Abstract] OR strength training[Title/Abstract] OR endurance exercise[Title/Abstract] OR yoga[Title/Abstract] OR Tai Chi[Title/Abstract] OR pilates[Title/Abstract] OR dance[Title/Abstract] OR walking[Title/Abstract] OR jogging[Title/Abstract] OR stretching[Title/Abstract] OR mind-body exercise[Title/Abstract])

AND

("Blood Pressure"[Mesh] OR (Pressure, Blood[Title/Abstract]) OR (Pulse Pressure[Title/Abstract]) OR (Pressure, Pulse[Title/Abstract]) OR (Diastolic Pressure[Title/Abstract]) OR (Pressure, Diastolic[Title/Abstract]) OR (Systolic Pressure[Title/Abstract]) OR (Pressures, Systolic[Title/Abstract]) OR (Pressure, Systolic[Title/Abstract])

AND

(Randomized Controlled Trial[Title/Abstract] OR RCT[Title/Abstract] OR randomized trial[Title/Abstract] OR controlled clinical trial[Title/Abstract] OR randomized study[Title/Abstract] OR randomized controlled trial[Title/Abstract] OR randomized clinical trial[Title/Abstract] OR RCTs[Title/Abstract] OR randomized clinical studies[Title/Abstract] OR intervention trial[Title/Abstract] OR randomized intervention[Title/Abstract] OR trial[Title/Abstract] OR experimental study[Title/Abstract])

1.2 Web of Science

TS=("chronic kidney disease" OR "CKD" OR "chronic renal disease" OR "chronic renal insufficiency" OR "chronic kidney failure" OR
"renal impairment" OR "diabetic nephropathy" OR "renal disease" OR
"end-stage renal disease" OR "ESRD" OR "hemodialysis" OR
"haemodialysis" OR "peritoneal dialysis" OR "kidney disease")

AND

TS=("Exercise" OR "Exercise Therapy" OR "Physical Fitness" OR
"Motor Activity" OR "Sports" OR "Resistance Training" OR "Yoga" OR "Tai Ji" OR "Dancing" OR "Walking" OR "Physical Exertion" OR "physical activity" OR "movement" OR

"fitness" OR "aerobic exercise" OR "resistance training"OR "high-intensity interval training" OR "HIIT" OR "strength training" OR "endurance exercise" OR "yoga" OR "tai chi" OR "pilates" OR "dance" OR "walking" OR "jogging" OR "stretching" OR "mind-body exercise")
AND
TS=("blood pressure" OR "systolic blood pressure" OR "systolic pressure" OR
"diastolic blood pressure" OR "diastolic pressure" OR
"hypertension" OR "arterial pressure" OR "antihypertensive" OR
"blood pressure control" OR "blood pressure reduction")
AND
TS=("randomized controlled trial" OR "randomised controlled trial" OR
"RCT" OR "randomized trial" OR "randomised trial" OR
"controlled clinical trial" OR "randomized study" OR
"randomised study" OR "randomized clinical trial" OR
"randomised clinical trial" OR "randomized intervention" OR
"intervention trial" OR "clinical trial")

1.3 Embase

('chronic kidney disease'/exp OR 'kidney disease'/exp OR
'chronic kidney disease':ti,ab,kw OR 'CKD':ti,ab,kw OR
'chronic renal disease':ti,ab,kw OR 'chronic renal insufficiency':ti,ab,kw OR
'chronic kidney failure':ti,ab,kw OR 'renal impairment':ti,ab,kw OR
'diabetic nephropathy':ti,ab,kw OR 'renal disease':ti,ab,kw OR
'end-stage renal disease':ti,ab,kw OR 'ESRD':ti,ab,kw OR
'kidney failure'/exp OR 'hemodialysis'/exp OR 'hemodialysis':ti,ab,kw OR
'haemodialysis':ti,ab,kw OR 'peritoneal dialysis'/exp OR
'peritoneal dialysis':ti,ab,kw)
AND
('Exercise'/exp OR 'Exercise Therapy':ti,ab OR 'Physical Fitness':ti,ab OR
'Motor Activity':ti,ab OR 'Sports':ti,ab OR 'Resistance Training':ti,ab OR
'Yoga':ti,ab OR 'Tai Ji':ti,ab OR 'Dancing':ti,ab OR 'Walking':ti,ab OR
'Physical Exertion':ti,ab OR 'Physical Activity':ti,ab OR 'Movement':ti,ab OR
'Fitness':ti,ab OR 'Aerobic Exercise':ti,ab OR 'Resistance Training':ti,ab OR 'high-intensity interval training':ti,ab OR 'HIIT':ti,ab OR 'Strength Training':ti,ab OR 'Endurance Exercise':ti,ab OR 'Yoga':ti,ab OR 'Tai Chi':ti,ab OR 'Pilates':ti,ab OR 'Dance':ti,ab OR 'Walking':ti,ab OR 'Jogging':ti,ab OR 'Stretching':ti,ab OR 'Mind-body Exercise':ti,ab)
AND
('blood pressure'/exp OR 'pulse pressure':ti,ab,kw OR 'diastolic blood pressure':ti,ab,kw OR 'pressure, diastolic':ti,ab,kw OR 'systolic blood pressure':ti,ab,kw OR 'pressures, systolic':ti,ab,kw OR 'pressure, systolic':ti,ab,kw OR 'elevated blood pressure':ti,ab,kw)
AND
('randomized controlled trial'/exp OR 'randomized controlled trial':ti,ab OR 'rct':ti,ab OR
'randomized trial':ti,ab OR 'controlled clinical trial':ti,ab OR 'randomized study':ti,ab OR
'randomized clinical trial':ti,ab OR 'rcts':ti,ab OR 'randomized clinical studies':ti,ab OR

'intervention trial':ti,ab OR 'randomized intervention':ti,ab OR 'trial':ti,ab OR 'experimental study':ti,ab)

1.4 Cochrane

#1 MeSH descriptor: [Renal Insufficiency, Chronic] explode all trees
#2 MeSH descriptor: [Kidney Diseases] explode all trees
#3 (chronic kidney disease):ti,ab,kw OR (CKD):ti,ab,kw OR
(chronic renal disease):ti,ab,kw OR (chronic renal insufficiency):ti,ab,kw OR
(chronic kidney failure):ti,ab,kw OR (renal impairment):ti,ab,kw OR
(diabetic nephropathy):ti,ab,kw OR (renal disease):ti,ab,kw OR
(end-stage renal disease):ti,ab,kw OR (ESRD):ti,ab,kw
#4 MeSH descriptor: [Renal Dialysis] explode all trees
#5 (hemodialysis):ti,ab,kw OR (haemodialysis):ti,ab,kw OR
(peritoneal dialysis):ti,ab,kw OR (dialysis):ti,ab,kw
#6 #1 OR #2 OR #3 OR #4 OR #5
#7 MeSH descriptor: [Exercise] explode all trees
#8 MeSH descriptor: [Exercise Therapy] explode all trees
#9 MeSH descriptor: [Motor Activity] explode all trees
#10 MeSH descriptor: [Sports] explode all trees
#11 MeSH descriptor: [Resistance Training] explode all trees
#12 (exercise):ti,ab,kw OR (physical activity):ti,ab,kw OR
(exercise training):ti,ab,kw OR (exercise therapy):ti,ab,kw OR
(motor activity):ti,ab,kw
#13 (aerobic exercise):ti,ab,kw OR (aerobic training):ti,ab,kw OR
(endurance exercise):ti,ab,kw
#14 (resistance training):ti,ab,kw OR (strength training):ti,ab,kw OR
(weight training):ti,ab,kw OR (muscle training):ti,ab,kw
#15 (blood flow restriction):ti,ab,kw OR (BFR training):ti,ab,kw OR
(occlusion training):ti,ab,kw
#16 (high-intensity interval training):ti,ab,kw OR (HIIT):ti,ab,kw OR
(interval training):ti,ab,kw OR (sprint interval training):ti,ab,kw
#17 (combined training):ti,ab,kw OR (combined exercise):ti,ab,kw OR
(concurrent training):ti,ab,kw
#18 MeSH descriptor: [Tai Ji] explode all trees
#19 (Tai Chi):ti,ab,kw OR (Tai-Chi):ti,ab,kw OR (Taichi):ti,ab,kw OR
(Taijiquan):ti,ab,kw OR (Tai Ji):ti,ab,kw OR (T'ai Chi):ti,ab,kw
#20 (Qigong):ti,ab,kw OR (Qi Gong):ti,ab,kw OR (Baduanjin):ti,ab,kw
#21 (mind-body exercise):ti,ab,kw OR (mind-body intervention):ti,ab,kw OR
(mind-body practice):ti,ab,kw
#22 MeSH descriptor: [Yoga] explode all trees
#23 (yoga):ti,ab,kw OR (yogic):ti,ab,kw OR (yoga therapy):ti,ab,kw OR
(pranayama):ti,ab,kw
#24 (Pilates):ti,ab,kw
#25 MeSH descriptor: [Walking] explode all trees

#26 (walking):ti,ab,kw OR (jogging):ti,ab,kw OR (running):ti,ab,kw OR (cycling):ti,ab,kw
#27 (stretching):ti,ab,kw OR (flexibility training):ti,ab,kw
#28 (aquatic exercise):ti,ab,kw OR (water exercise):ti,ab,kw OR (hydrotherapy):ti,ab,kw OR (swimming):ti,ab,kw
#29 (intradialytic exercise):ti,ab,kw OR (intradialytic training):ti,ab,kw OR (intra-dialytic):ti,ab,kw
#30 (home-based exercise):ti,ab,kw OR (home-based training):ti,ab,kw
#31 (isometric exercise):ti,ab,kw OR (isometric training):ti,ab,kw OR (isometric contraction):ti,ab,kw
#32 #7 OR #8 OR #9 OR #10 OR #11 OR #12 OR #13 OR #14 OR #15 OR #16 OR #17 OR #18 OR #19 OR #20 OR #21 OR #22 OR #23 OR #24 OR #25 OR #26 OR #27 OR #28 OR #29 OR #30 OR #31
#33 MeSH descriptor: [Blood Pressure] explode all trees
#34 (blood pressure):ti,ab,kw OR (systolic blood pressure):ti,ab,kw OR (systolic pressure):ti,ab,kw OR (diastolic blood pressure):ti,ab,kw OR (diastolic pressure):ti,ab,kw
#35 MeSH descriptor: [Hypertension] explode all trees
#36 (hypertension):ti,ab,kw OR (arterial pressure):ti,ab,kw OR (antihypertensive):ti,ab,kw OR (blood pressure control):ti,ab,kw OR (blood pressure reduction):ti,ab,kw OR (elevated blood pressure):ti,ab,kw
#37 #33 OR #34 OR #35 OR #36
#38 #6 AND #32 AND #37
#39 #38 in Trials

2. Encoding example

MET values were assigned from the 2024 Adult Compendium of Physical Activities according to the reported exercise modality, intensity, and session description. When a report did not provide enough detail for an exact compendium code, the closest conservative activity category was selected. Broad modalities such as tai chi, yoga, resistance training, and combined aerobic-resistance training may include within-category variation; therefore, the calculated MET-min/week values are standardized exposure estimates rather than patient-specific measures of relative exercise intensity.

- **Agent:** The agent level.
- **MET_min:** The energy expenditure per min in the study.
- **Min_session**： The duration of a single exercise intervention.
- **Frequency:** The number of days participants engaged in physical activity.
- **Exact_dose:** The exact estimated METs accumulated per week by participants.
- **Residual_dose:** The difference between the exact dose and the approximated dose.
- **Dose:** The dose group by approximation.
- **Y:** Standardised mean difference
- **SE:** Standard error of the mean.
- **CG:** Control group

study ID	agent	MET_min	min_session	MET_session	frequency	exact_dose	residual_dose	dose	Average adherence rate (%)	n
1	RT	3.5	90	315	2	504	4	500	80%	20
1	CG	0	0	0	0	0	0	0	0	20

3. Table S1. Study characteristics

Study	Interventions (n)	length （week）	Age (mean±sd)	Region	Chronic Kidney Disease type	Frequency (times/week)	Duration (min)	Exercise Intensity	RRT
Aoike et al. 2015 [47]	AE(14)	12	55.9(7.7)	Brazil	CKD stages 3-4	3	30min	Medium intensity	No
	CG(15)	12	54.3(8.7)	Brazil	CKD stages 3-4	NR	NR	NR	No
Aoike et al. 2018 [48]	AE(12)	24	56(8.3)	Brazil	CKD stages 3-4	3	30min	Medium intensity	No
	AE(13)	24	56.3(7.9)	Brazil	CKD stages 3-4	3	30min	Medium intensity	No
	CG(15)	24	54.3(8.7)	Brazil	CKD stages 3-4	NR	NR	NR	No
Barcellos et al. 2018 [49]	AE-RT(58)	16	65(1.2)	Brazil	CKD stages 2-4	3	60min	NR	No
	CG(51)	16	65.1(1.3)	Brazil	CKD stages 2-4	NR	NR	NR	No
Cooke et al . 2018 [50]	AE(10)	16	58.2(17.2)	Canada	Stage 5 CKD	3	42.6min	Moderate to high intensity	Yes
	CG(10)	16	52.5(15.4)	Canada	Stage 5 CKD	NR	NR	NR	Yes
Corrêa et al . 2021 [51]	RT(30)	26	58(9)	Brazil	Stage 2 CKD	3	30min	Moderate to high intensity	No
	RT(30)	26	60(8)	Brazil	Stage 2 CKD	3	30min	Low to moderate intensity	No
	CG(30)	26	57(6)	Brazil	Stage 2 CKD	NR	NR	NR	No
de Araújo et al . 2023 [52]	RT(16)	22	57.94(2.74)	Brazil	Stage 2 CKD	3	40min	Mild to moderate to severe	No
	CG(15)	22	58.07(5.22)	Brazil	Stage 2 CKD	NR	NR	NR	No
Deligiannis et al . 1999 [53]	AE(16)	26	46.4(13.9)	Greece	Stage 5 CKD	3	90min	Medium intensity	Yes
	AE(10)	26	51.4(12.5)	Greece	Stage 5 CKD	5	30min	Low-intensity	Yes
	CG(12)	26	50.2(7.9)	Greece	Stage 5 CKD	NR	NR	NR	Yes
Greenwood et al . 2015 [54]	AE-RT(8)	52	53.8(13.5)	UK	CKD stages 3-4	3	40min	High strength	No
	CG(10)	52	53.3(12.9)	UK	CKD stages 3-4	NR	NR	NR	No
Headley et al . 2012 [55]	AE-RT(10)	48	57.5(11.5)	USA	CKD stages 2-4	3	45min	Medium intensity	No
	CG(11)	48	52.5(10.6)	USA	CKD stages 2-4	NR	NR	NR	No
Headley et al . 2014 [56]	AE(25)	16	58(8)	USA	Stage 3 CKD	3	55min	Medium intensity	No
	CG(21)	16	57.1(9)	USA	Stage 3 CKD	NR	NR	NR	No
Howden et al . 2013 [57]	AE-RT(36)	52	60.2(9.7)	Australia	CKD stages 3-4	2-3	20-30min	Medium intensity	No
	CG(36)	52	62(8.4)	Australia	CKD stages 3-4	NR	NR	NR	No
Huang et al . 2020 [58]	AE-RT(16)	24	43.81(10.25)	China	Stage 5 CKD	3	40min	Medium intensity	Yes
	CG(16)	24	37.63(10.31)	China	Stage 5 CKD	NR	NR	NR	Yes
Huppertz et al .2020 [59]	AE(56)	52	58.8(9.7)	Australia	CKD stages 3-4	2-3	75min	Medium intensity	No
	CG(57)	52	62.1(8.6)	Australia	CKD stages 3-4	NR	NR	NR	No
Jeong et al. 2019 [60]	AE(29)	52	53.7(11.4)	USA	Stage 5 CKD	3	45min	Medium intensity	Yes
	CG(37)	52	56.6(13)	USA	Stage 5 CKD	NR	NR	NR	Yes
Kirkman et al . 2019 [61]	AE(16)	12	55(13)	USA	CKD stages 3-5	3	45min	Moderate to high strength	No
	CG(15)	12	62(9)	USA	CKD stages 3-5	NR	NR	NR	No
Koh et al . 2010 [62]	AE(15)	26	52.3(10.9)	Australia	Stage 5 CKD	3	45min	Medium intensity	Yes
	AE(15)	26	52.1(13.6)	Australia	Stage 5 CKD	3	45min	Medium intensity	Yes
	CG(16)	26	51.3(14.4)	Australia	Stage 5 CKD	NR	NR	NR	Yes
Leehey et al . 2009 [63]	AE(7)	24	NR	USA	CKD stages 2-4	3	35min	Medium intensity	No
	CG(4)	24	NR	USA	CKD stages 2-4	NR	NR	NR	No
Leehey et al . 2016 [64]	AE-RT(14)	52	65.4(8.7)	USA	CKD stages 2-4	3	45min	Mild to moderate intensity	No

	CG(18)	52	66.6(7.5)	USA	CKD stages 2-4	NR	NR	NR	No
Marrone et al .2024 [65]	AE-RT(10)	12	58.44(8.25)	Italy	CKD stages 2-4	3	60min	Moderate to high intensity	No
	CG(10)	12	65.5(4.43)	Italy	CKD stages 2-4	NR	NR	NR	No
Molsted et al .2004 [66]	AE-RT(11)	22	59(24.44)	Denmark	Stage 5 CKD	2	60min	Medium intensity	Yes
	CG(9)	22	48(24.44)	Denmark	Stage 5 CKD	NR	NR	NR	Yes
Shi et al .2014 [67]	Mind-body(11)	12	69.5(6.9)	China	Stage 3b CKD	3-5	30min	Medium intensity	No
	CG(10)	12	68.4(8.7)	China	Stage 3b CKD	NR	NR	NR	No
Suvannarot et al . 2025 [68]	AE(31)	12	66.45(7.91)	Thailand	CKD stages 2-3	3	30min	Low-intensity	No
	CG(33)	12	69.03(6.96)	Thailand	CKD stages 2-3	NR	NR	NR	No
van Vilsteren et al . 2005 [69]	AE-RT(47)	12	52(15)	Netherlands	Stage 5 CKD	2-3	50min	Low to moderate intensity	Yes
	CG(43)	12	58(16)	Netherlands	Stage 5 CKD	NR	NR	NR	Yes
Watson et al . 2018 [22]	AE(18)	12	63(9.63)	UK	CKD stages 3b-5	3	30min	Medium to high intensity	No
	AE-RT(18)	12	63(13.33)	UK	CKD stages 3b-5	2	25min	High strength	No
Pandey et al .2017 [70]	Mind-body(25)	26	43.32(13.32)	India	CKD	5	40-60min	NR	Yes
	CG(25)	26	44.4(13.68)	India	CKD	NR	NR	NR	Yes
Nair et al .2026 [71]	Mind-body(38)	12	54.18(4.6)	India	CKD stages 3-4	5	60min	NR	No
	CG(34)	12	53.2(6)	India	CKD stages 3-4	NR	NR	NR	No

Note:RRT:Renal replacement therapy;AE:Aerobic Exercise；,RT:Resistance Training；,AE-RT:Combined Aerobic and Resistance Training；,CG:control group；CKD:Chronic Renal Failure Reference numbers correspond to the reference list in the main manuscript.

Since there is no unified description of exercise intensity across different articles, the "Exercise Intensity" column in this table represents a qualitative summary of the original intensity indicators, adhering to the following principles: (1) If the author has already provided a qualitative classification (e.g., "moderate intensity"), it is directly adopted; (2) If the literature only provides objective indicators (e.g.,%HRmax, RPE), they are converted based on general consensus in exercise science (e.g., 50-63% HRmax ≈ low intensity; RPE 12-16 ≈ moderate intensity).

4. Risk of bias

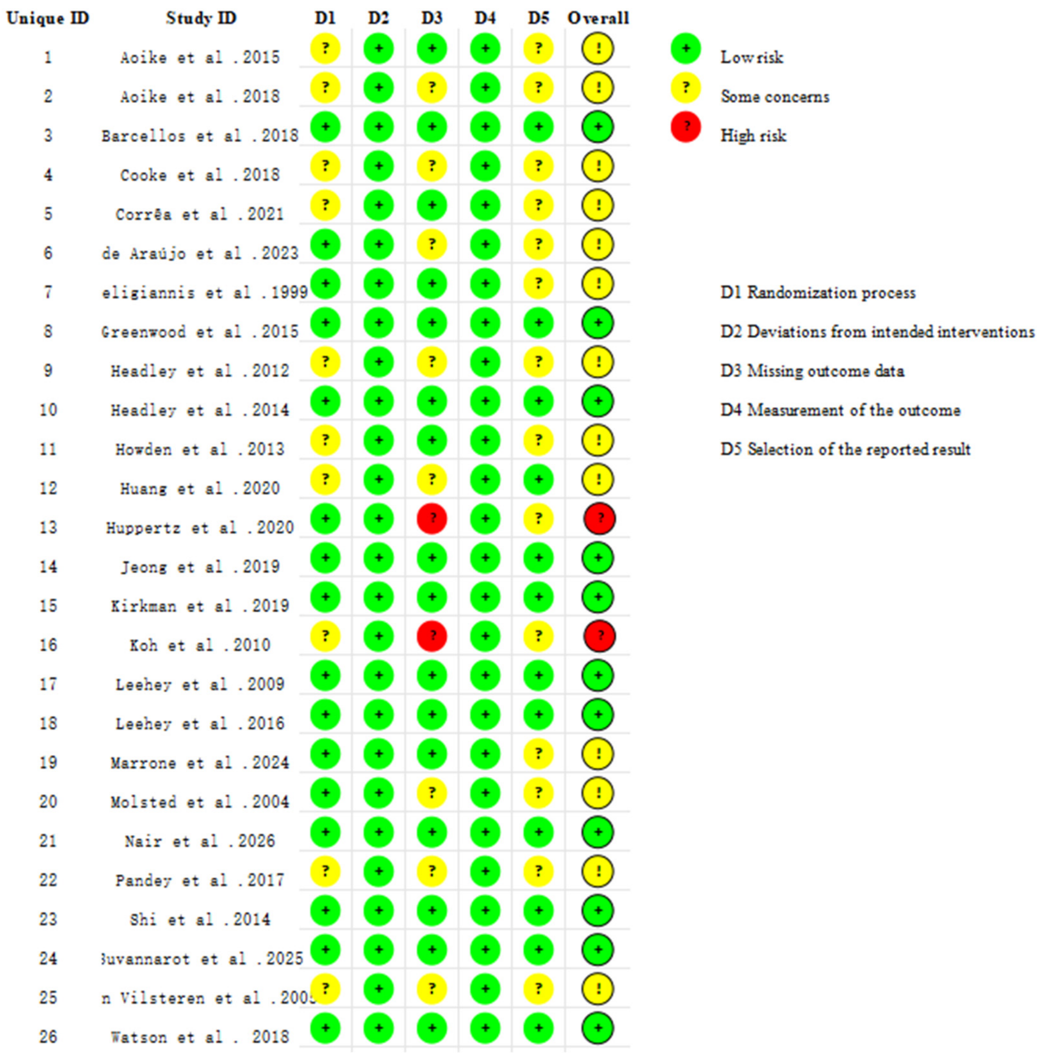

Figure S1. The detail of risk of bias.

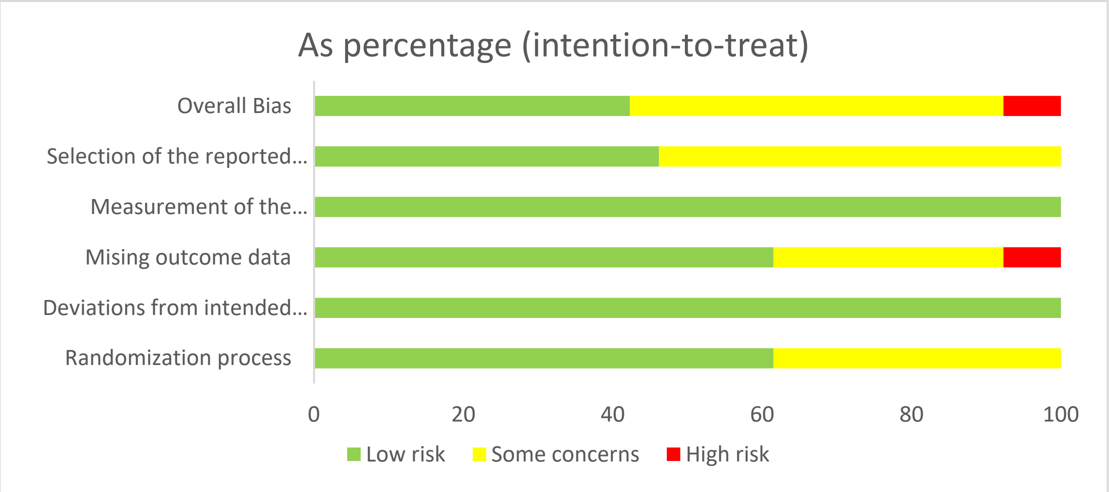

Figure S2. The summary of risk of bias.

5. Dose-response network meta-analyses

5.1 Connectivity

Connectivity is a fundamental assumption in network meta-analysis (NMA). Insufficient connectivity, often resulting from limited direct comparisons, can reduce statistical power and bias results. In our study, connectivity was evaluated at both the intervention (Figure S3) and dose levels (Figue 2), and no evidence of network disconnection was observed.

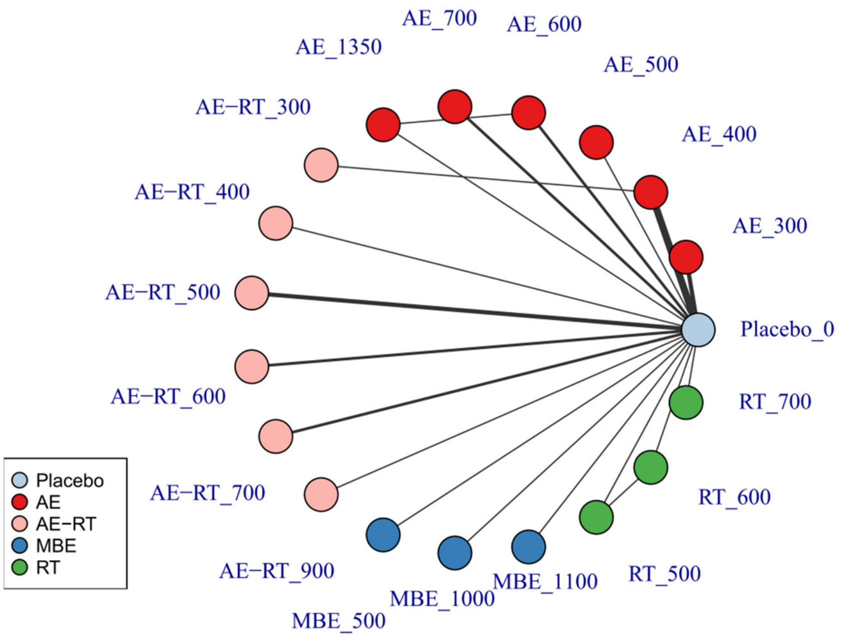

Figure S3. Dose-level network (systolic blood pressure).

Line thickness represents the number of studies available for comparison. The first value represents the specific intervention, and the second value corresponds to the dose (METs - min/week).

AE: Aerobic Exercise; AE - RT: Combined Aerobic and Resistance Training; RT: Resistance Training;MBE:Mind-body exercise; CG: Control group

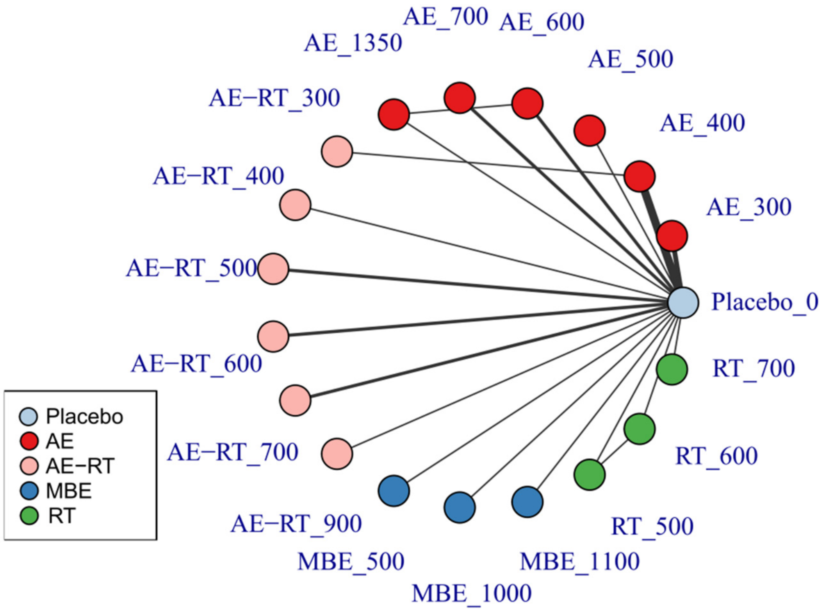

Figure S4. Dose-level network (diastolic blood pressure)

Line thickness represents the number of studies available for comparison. The first value represents the specific intervention, and the second value corresponds to the dose (METs-min/week).

AE: aerobic exercise; AE-RT: combined aerobic and resistance training; RT: resistance training; MBE: mind-body exercise; CG: control group

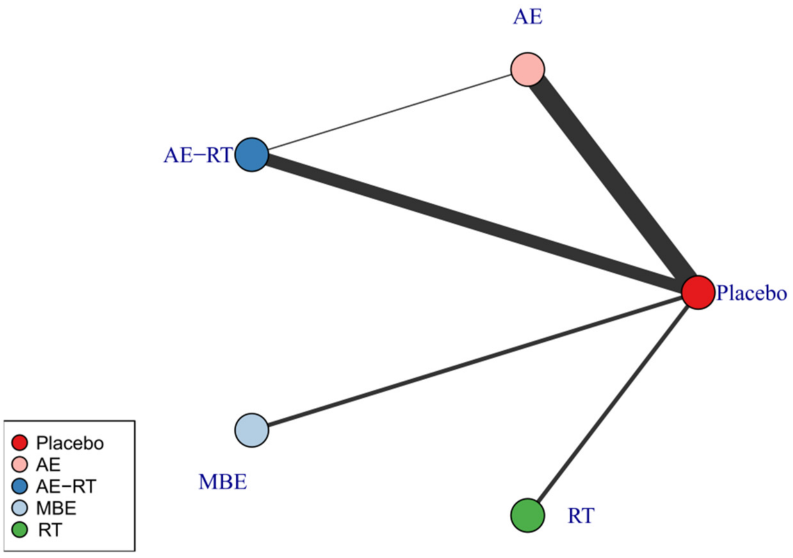

Figure S5. Agent-level network (systolic blood pressure).

Line thickness represents the number of studies available for comparison.

AE: Aerobic Exercise; AE-RT: Combined Aerobic and Resistance Training; RT: Resistance Training;MBE:Mind-body exercise; CG: Control group

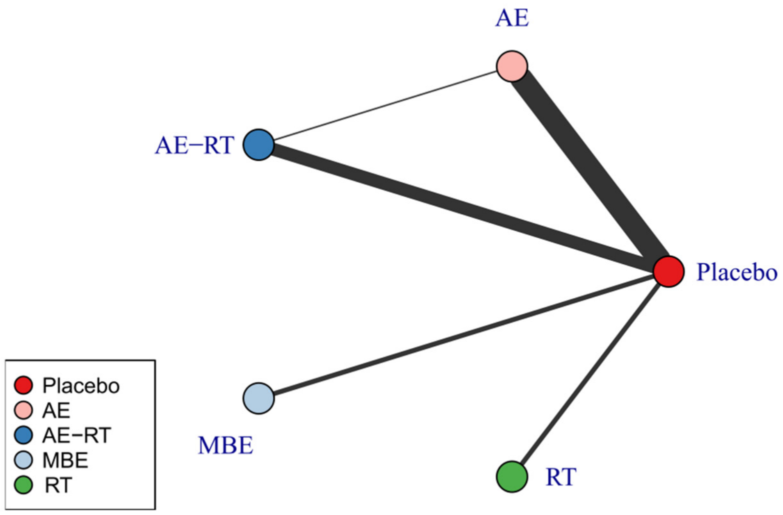

Figure S6. Agent-level network (diastolic blood pressure).

Line thickness represents the number of studies available for comparison.

AE: Aerobic Exercise; AE-RT: Combined Aerobic and Resistance Training; RT: Resistance Training;MBE:Mind-body exercise; CG: Control group

5.2 Consistency

We performed a consistency analysis by comparing the consistency model with the unrelated mean effects (UME) model. In practice, we compared the number of estimated parameters, deviance information criterion (DIC), residual deviance, and residual standard deviation. These parameters showed acceptable agreement between models (Tables S2 and S3).

Table S2. Systolic blood pressure Comparison of consistency and UME model fit.

Model	PD	Deviance	Residual deviance	DIC	SD
Consistent	41.8	292.022	55.388	341.0	4.460

UME	42.7	292.307	55.672	334.6	4.588

Note: UME = unrelated mean effects model; DIC = deviance information criterion; PD = effective number of parameters; SD = standard deviation

Table S3. Diastolic blood pressure Comparison of consistency and UME model fit.

Model	PD	Deviance	Residual deviance	DIC	SD
Consistent	42.7	235.057	52.550	278.0	3.745
UME	44.1	235.050	52.543	279.7	3.861

Note: UME = unrelated mean effects model; DIC = deviance information criterion; PD = effective number of parameters; SD = standard deviation

5.3 Transitivity

Network meta-analyses (NMAs) rely on the assumption of transitivity, meaning that direct and indirect estimates are sufficiently comparable after accounting for expected random-effects variation. We evaluated potential inconsistency at the treatment level using MBNMA node-splitting methods, which separate direct and indirect evidence for each contrast and compare their agreement. Results of this assessment are presented in Tables S4 and S5.

Table S4. Systolic blood pressure Node-splitting analysis of consistency.

Comparison	p-value	Median	2.50%	97.50%
AE-RT_350 vs AE_400	0.482			
-> direct		7.302	-6.571	20.723
-> indirect		3.536	-1.500	8.570
-> MBNMA		3.784	-0.803	8.425
RT_700 vs Placebo_0	0.183			
-> direct		-5.686	-14.157	2.350
-> indirect		-14.316	-19.417	-8.999
-> MBNMA		-12.182	-16.824	-6.271
RT_600 vs Placebo_0	0.654			
-> direct		-13.817	-21.093	-7.037
-> indirect		-11.010	-16.926	-3.761
-> MBNMA		-12.013	-16.644	-6.158
RT_500 vs Placebo_0	0.435			
-> direct		-14.116	-21.527	-6.733

-> indirect		-9.033	-15.444	-0.948
-> MBNMA		-11.763	-16.323	-6.032
Mind-body_1100 vs Placebo_0	0.116			
-> direct		-5.685	-13.549	2.008
-> indirect		-17.908	-25.679	-9.948
-> MBNMA		-11.544	-18.028	-5.795
Mind-body_1000 vs Placebo_0	0.203			
-> direct		-18.131	-27.261	-9.040
-> indirect		-7.439	-14.971	-0.515
-> MBNMA		-11.464	-17.899	-5.761
Mind-body_500 vs Placebo_0	0.425			
-> direct		-16.988	-33.823	-0.510
-> indirect		-10.119	-16.508	-3.714
-> MBNMA		-10.729	-16.844	-5.322
AE-RT__900 vs Placebo_0	0.306			
-> direct		4.397	-13.500	21.867
-> indirect		-2.915	-7.272	1.472
-> MBNMA		-2.618	-6.631	1.725
AE-RT__700 vs Placebo_0	0.556			
-> direct		-3.290	-14.394	8.309
-> indirect		-2.249	-6.662	2.146
-> MBNMA		-2.567	-6.447	1.672
AE-RT__600 vs Placebo_0	0.589			
-> direct		-4.370	-12.038	2.593
-> indirect		-1.176	-6.524	3.634
-> MBNMA		-2.516	-6.327	1.648
AE-RT_500 vs Placebo_0	0.359			
-> direct		1.068	-5.695	8.167
-> indirect		-4.002	-8.784	0.862
-> MBNMA		-2.467	-6.215	1.611
AE-RT_400 vs Placebo_0	0.066			
-> direct		-18.184	-33.579	-2.723
-> indirect		-1.468	-4.996	2.461
-> MBNMA		-2.381	-6.056	1.599
AE_1350 vs Placebo_0	0.381			
-> direct		-10.877	-21.375	0.511
-> indirect		-6.532	-10.002	-2.862
-> MBNMA		-6.792	-10.204	-3.389
AE_700 vs placebo_0	0.123			
-> direct		-0.206	-7.091	6.907
-> indirect		-8.062	-11.311	-4.512

-> MBNMA		-6.465	-9.522	-3.236
AE_600 vs placebo_0	0.797			
-> direct		-6.537	-13.176	0.892
-> indirect		-6.366	-10.223	-2.337
-> MBNMA		-6.367	-9.393	-3.165
AE_500 vs placebo_0	0.394			
-> direct		-4.327	-18.090	8.955
-> indirect		-6.415	-9.534	-2.793
-> MBNMA		-6.224	-9.178	-3.109
AE_400 vs placebo_0	0.132			
-> direct		-10.821	-15.605	-5.763
-> indirect		-4.491	-7.629	-0.805
-> MBNMA		-6.028	-8.907	-2.975
AE_300 vs placebo_0	0.373			
-> direct		-1.796	-10.423	-6.789
-> indirect		-6.232	-9.414	-3.163
-> MBNMA		-5.734	-8.573	-2.778

Note: The first value represents the specific intervention, and the second value corresponds to the dose MET-min/week. AE: Aerobic Exercise; AE-RT: Combined Aerobic and Resistance Training; RT: Resistance Training;

Table S5. Diastolic blood pressure Node-splitting analysis of consistency.

**Comparison**	**p-value**	**Median**	**2.50%**	**97.50%**
AE-RT_300 vs AE_400	0.583			
-> direct		3.866	-4.802	12.528
-> indirect		1.984	-1.781	6.231
-> MBNMA		2.348	-1.255	5.951
RT_700 vs Placebo_0	0.030			
-> direct		--1.125	-9.189	5.891
-> indirect		-14.896	-19.336	-10.775
-> MBNMA		-11.008	-15.866	-5.183
RT_600 vs Placebo_0	0.368			
-> direct		-14.605	-20.928	-8.773
-> indirect		-9.648	-14.951	-3.644
-> MBNMA		-10.842	-15.631	-5.101
RT_500 vs Placebo_0	0.302			
-> direct		-13.950	-20.999	-6.566
-> indirect		-6.893	-13.614	0.539
-> MBNMA		-10.602	-15.286	-4.989
Mind-body_1100 vs Placebo_0	0.765			
-> direct		-10.068	-17.537	-2.190
-> indirect		-8.089	-15.069	-1.099

-> MBNMA		-9.041	-14.031	-4.049
Mind-body_1000 vs Placebo_0	0.551			
-> direct		-6.328	-14.437	1.993
-> indirect		-10.428	-16.959	-4.050
-> MBNMA		-8.985	-13.916	-4.042
Mind-body_500 vs Placebo_0	0.558			
-> direct		-10.912	-22.789	0.630
-> indirect		-7.897	-13.178	-2.768
-> MBNMA		-8.346	-13.129	-3.787
AE-RT__900 vs Placebo_0	0.254			
-> direct		5.763	-8.253	19.349
-> indirect		-2.280	-5.415	1.257
-> MBNMA		-1.888	-5.027	1.720
AE-RT__700 vs Placebo_0	0.603			
-> direct		-1.783	-10.068	6.879
-> indirect		-1.876	-5.083	1.940
-> MBNMA		-1.853	-4.924	1.670
AE-RT__600 vs Placebo_0	0.241			
-> direct		-5.168	-10.310	0.078
-> indirect		0.066	-3.703	3.829
-> MBNMA		-1.821	-4.851	1.637
AE-RT_500 vs Placebo_0	0.701			
-> direct		-0.623	-6.607	5.427
-> indirect		-2.217	-5.799	2.129
-> MBNMA		-1.779	-4.755	1.607
AE-RT_400 vs Placebo_0	0.287			
-> direct		4.535	-8.325	17.733
-> indirect		-2.006	-5.000	1.288
-> MBNMA		-1.727	-4.648	1.551
AE_1350 vs Placebo_0	0.379			
-> direct		-7.876	-15.262	-0.804
-> indirect		-4.127	-6.936	-1.047
-> MBNMA		-4.480	-7.273	-1.571
AE_700 vs placebo_0	0.191			
-> direct		-0.246	-5.500	5.217
-> indirect		-5.486	-8.052	-5.687
-> MBNMA		-4.269	-6.749	-1.491
AE_600 vs placebo_0	0.651			
-> direct		-5.212	-10.815	0.694
-> indirect		-3.791	-6.968	-0.693
-> MBNMA		-4.197	-6.611	-1.468

AE_500 vs placebo_0	0.307			
-> direct		0.433	-10.983	11.012
-> indirect		-4.309	-6.821	-1.594
-> MBNMA		-4.092	-6.425	-1.437
AE_400 vs placebo_0	0.223			
-> direct		-7.325	-11.538	-2.932
-> indirect		-3.072	-5.670	-0.176
-> MBNMA		-3.964	-6.266	-1.410
AE_300 vs placebo_0	0.443			
-> direct		-1.397	-7.366	5.028
-> indirect		-4.021	-6.640	-1.538
-> MBNMA		-3.766	-5.998	-1.315

Note: The first value represents the specific intervention, and the second value corresponds to the dose MET-min/week. AE: Aerobic Exercise; AE-RT: Combined Aerobic and Resistance Training; RT: Resistance Training;

5.4 Model selection (systolic blood pressure as an example)

For model selection, we fitted several dose-response functions and selected the primary model according to model-fit criteria, including DIC, residual deviance, residual standard deviation, and model complexity. The quadratic function model showed the lowest DIC among the tested models and was therefore selected as the primary model (Tables S6 and S7). The resulting dose-response peaks should be interpreted as model-estimated values within the observed evidence base, not as validated clinical thresholds.

Table S6. Systolic blood pressure Models fit comparison

Model	DIC	SD	Deviance	Residual deviance	pD
Emax (random treatment effects)	332.0	2.917	293.437	56.802	37.7
Restricted cubic spline (random treatment effects; 3 knots)	344.3	0.715	317.192	80.558	49.6
Log Linear (random treatment effect)	332.8	6.159	314.678	78.044	39.4
Non-parametric monotonically up (random treatment effects)	333.3	4.782	310.098	73.464	42.8

Exponential dose-response function (random treatment effects)	341.6	0.771	315.759	79.125	48.4
Quadratic function (random treatment effects)	331.7	6.976	313.881	77.246	41.5
Spline dose-response functions (random treatment effects)	332.2	5.160	311.192	74.557	41.3
Fractional polynomial dose-response function (random treatment effects)	341.8	0.885	316.154	79.520	49.3

Note: DIC: deviance information criterion; pD: effective number of parameters; SD: standard deviation.

Table S7. Diastolic blood pressure Models fit comparison

Model	DIC	SD	Deviance	Residual deviance	pD
Emax (random treatment effects)	277.5	3.056	235.208	52.701	41.8
Restricted cubic spline (random treatment effects; 3 knots)	277.7	4.084	254.917	72.411	41.6
Log Linear (random treatment effect)	277.9	3.132	236.230	53.723	41.3
Non-parametric monotonically up (random treatment effects)	276.3	3.924	254.446	71.939	40.7
Exponential dose-response function (random treatment effects)	284.8	0.650	236.252	53.745	48.6
Quadratic function (random treatment effects)	273.5	3.754	235.247	52.741	43.7

Spline dose-response functions (random treatment effects)	275.7	4.052	254.875	72.368	40.4
Fractional polynomial dose-response function (random treatment effects)	284.8	0.710	235.896	53.389	49.1

We also validated the fit of the quadratic function model to the data. Figure S7 presents the results of the “Splict” network meta-analysis at different doses of various exercises on systolic blood pressure. This step aimed to explore the dose–response pattern and to assess which functional form best fits the data, thereby informing the selection of a model for the subsequent MBNMA. The model that best matches the pattern shown in Figure S7 should be selected accordingly.

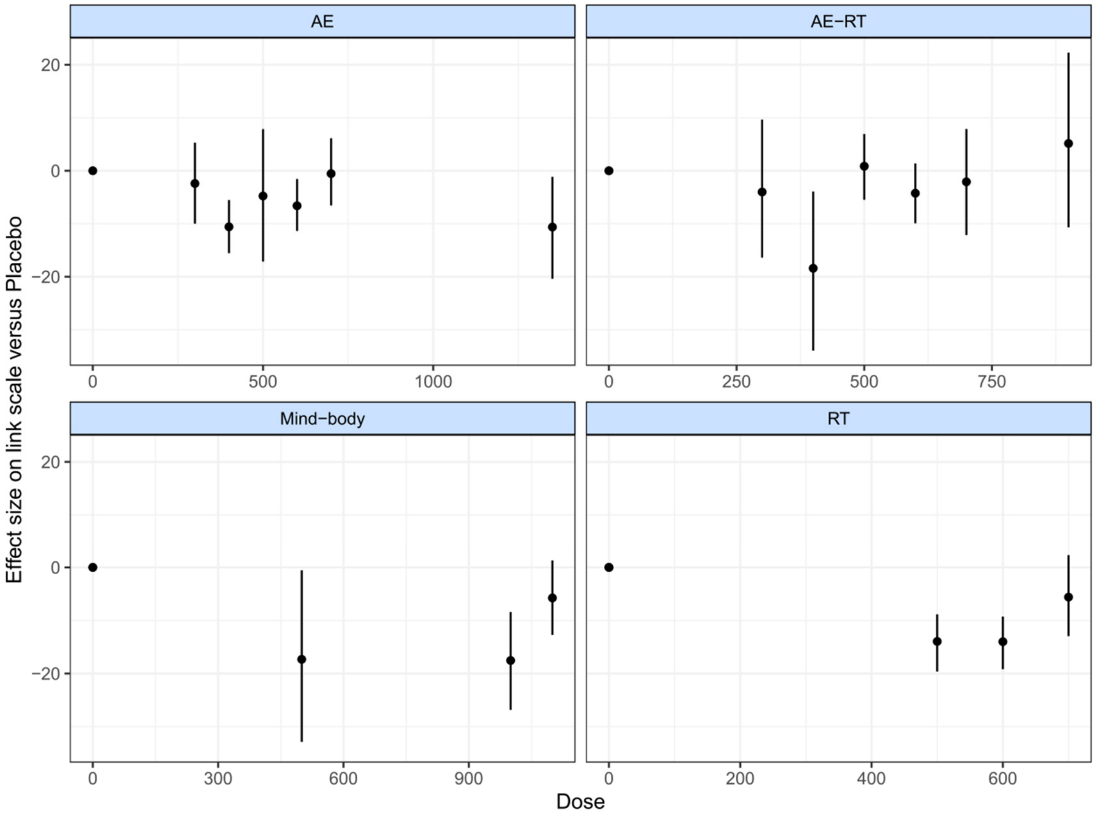

Figure S7. "Split" network meta-analysis of different exercise interventions on systolic blood pressure.

The black dots represent the pooled effect size from the meta-analysis at that dose, and the black vertical lines represent the range of the 95% CrI. AE: Aerobic Exercise; AE-RT: Combined Aerobic and Resistance Training; RT: Resistance Training.

We observed that the trend of the model curve is generally consistent with the data points from the "Split" network meta-analysis, suggesting a good fit between the model and the data.

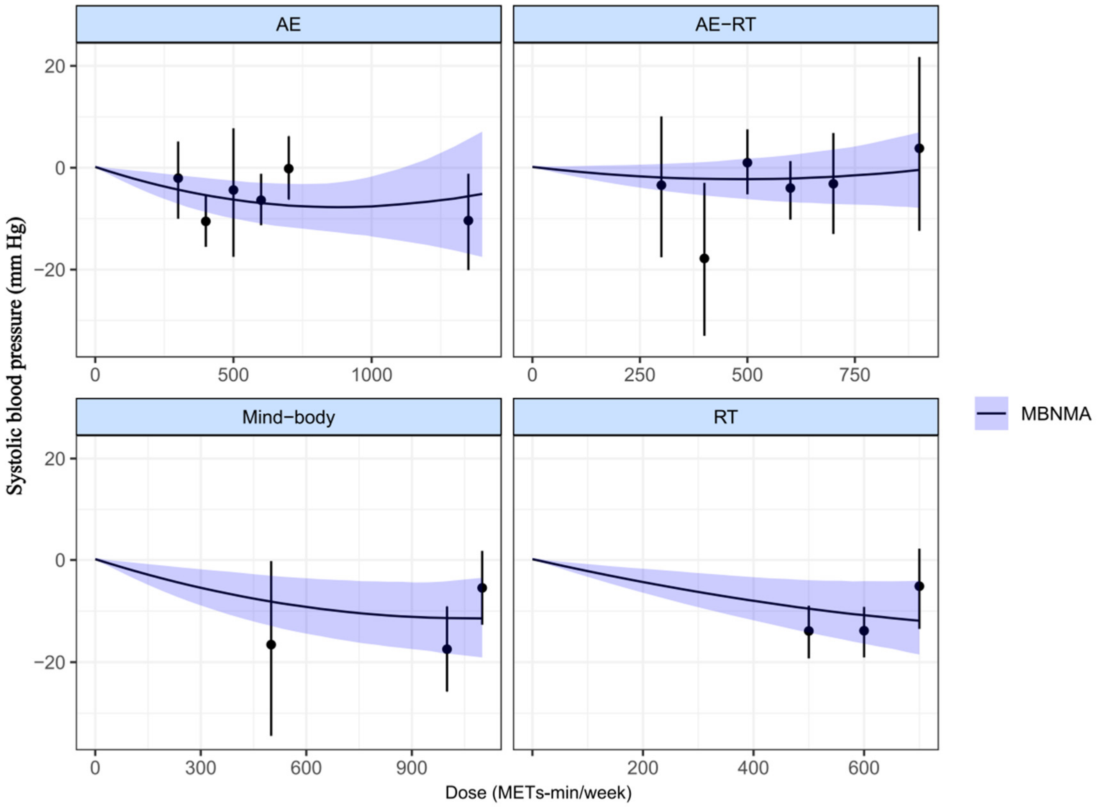

Figure S8. Dose–response relationship between different exercise (METs-min/week) and systolic blood pressure (quadratic function).

The black dots represent the pooled effect size from the meta-analysis at that dose, while the blue region represents the 95% CrI range. AE: Aerobic Exercise; AE-RT: Combined Aerobic and Resistance Training; RT: Resistance Training.

5.5 Subgroup analysis

5.5.1 Systolic blood pressure (baseline blood pressure ≥140 mmHg)

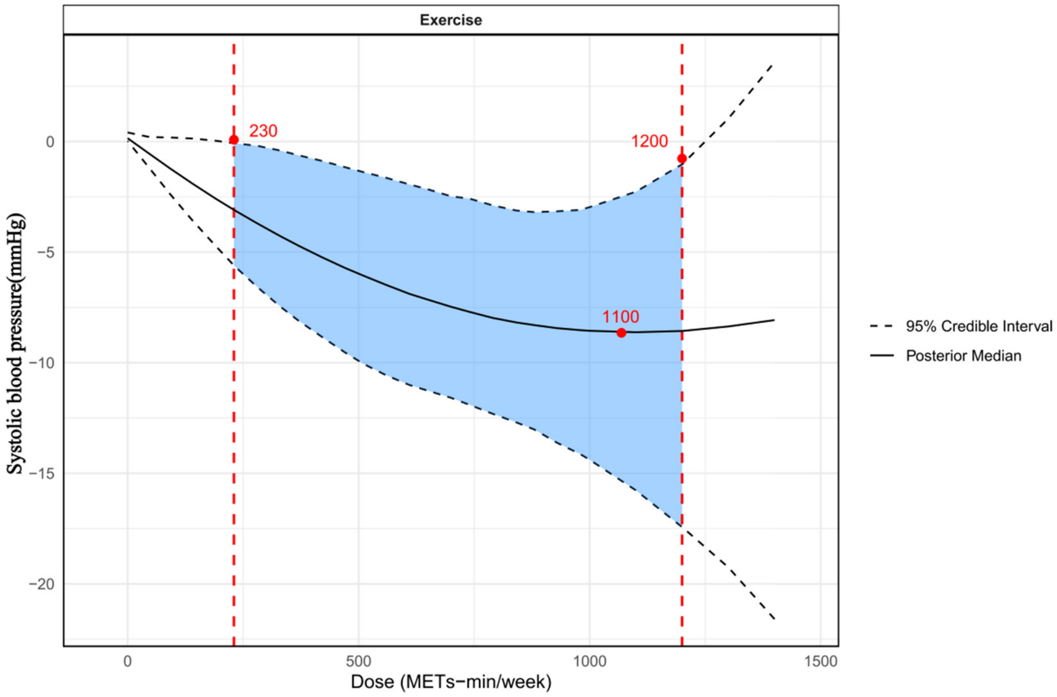

Figure S9. Dose–response relationship between exercise and systolic blood pressure (≥140 mm Hg) at baseline.

The red dashed lines represent the model-estimated lower and upper exposure values associated with statistically significant reductions (230 and 1200 MET-min/week), while the red shaded area indicates the statistically significant region. Red dots represent the exposure values associated with the greatest predicted reductions within the modelled range (1100 MET-min/week).

5.5.2 Systolic blood pressure (baseline blood pressure <140 mmHg)

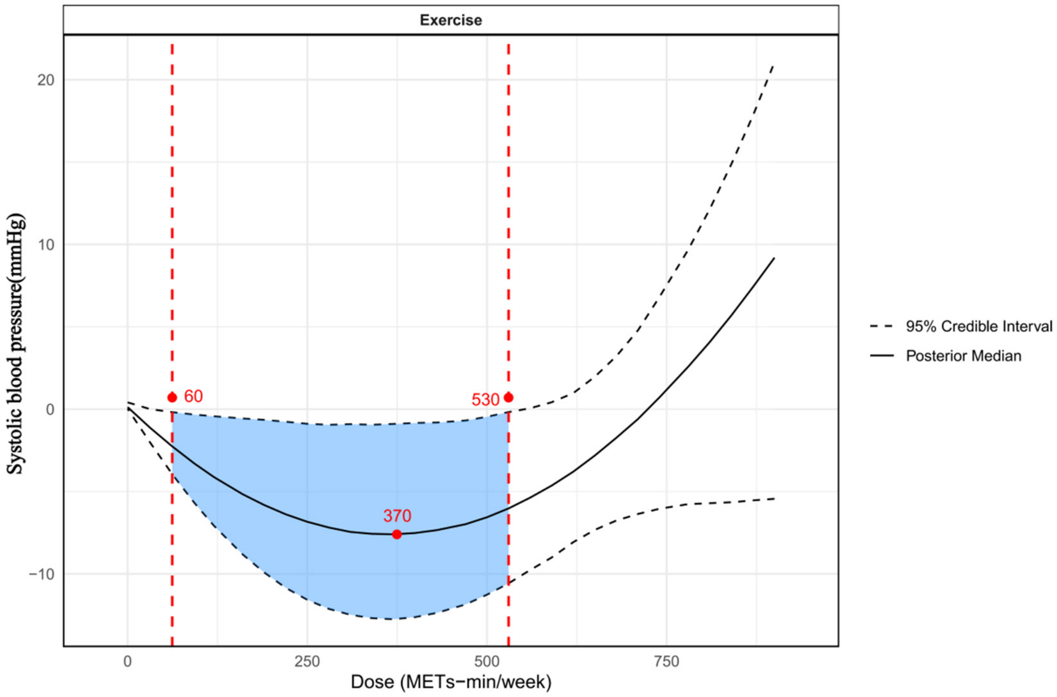

Figure S10. Dose–response relationship between exercise and systolic blood pressure (<140 mm Hg) at baseline.

The red dashed lines represent the model-estimated lower and upper exposure values associated with statistically significant reductions (60 and 530 MET-min/week), while the red shaded area indicates the statistically significant region. Red dots represent the exposure values associated with the greatest predicted reductions within the modelled range (370 MET-min/week).

5.5.3 Systolic blood pressure (non-dialysis population)

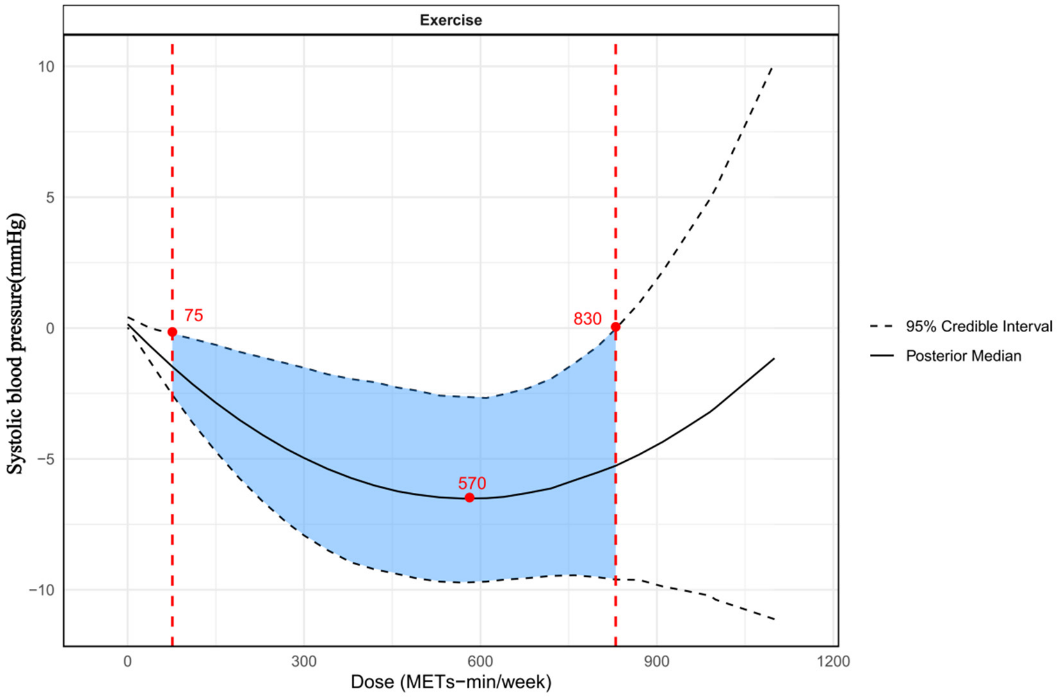

Figure S11. Dose–response relationship between exercise and systolic blood pressure (non-dialysis population).

The red dashed lines represent the model-estimated lower and upper exposure values associated with statistically significant reductions (75 and 830 MET-min/week), while the red shaded area indicates the statistically significant region. Red dots represent the exposure values associated with the greatest predicted reductions within the modelled range (570 MET-min/week).

5.5.4 Systolic blood pressure (dialysis population)

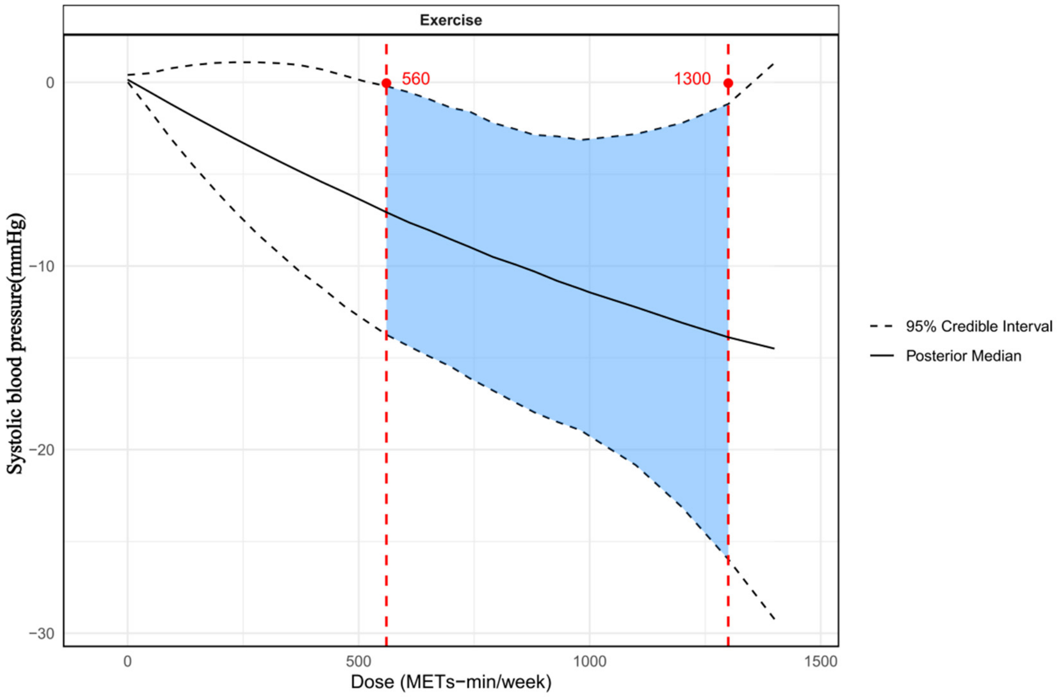

Figure S12. Dose–response relationship between exercise and systolic blood pressure (dialysis population).

The red dashed lines represent the model-estimated lower and upper exposure values associated with statistically significant reductions (560 and 1300 MET-min/week), while the red shaded area indicates the statistically significant region. Red dots represent the exposure values associated with the greatest predicted reductions within the modelled range (1300 MET-min/week).

5.5.5 Systolic blood pressure (intervention length ＞12 weeks)

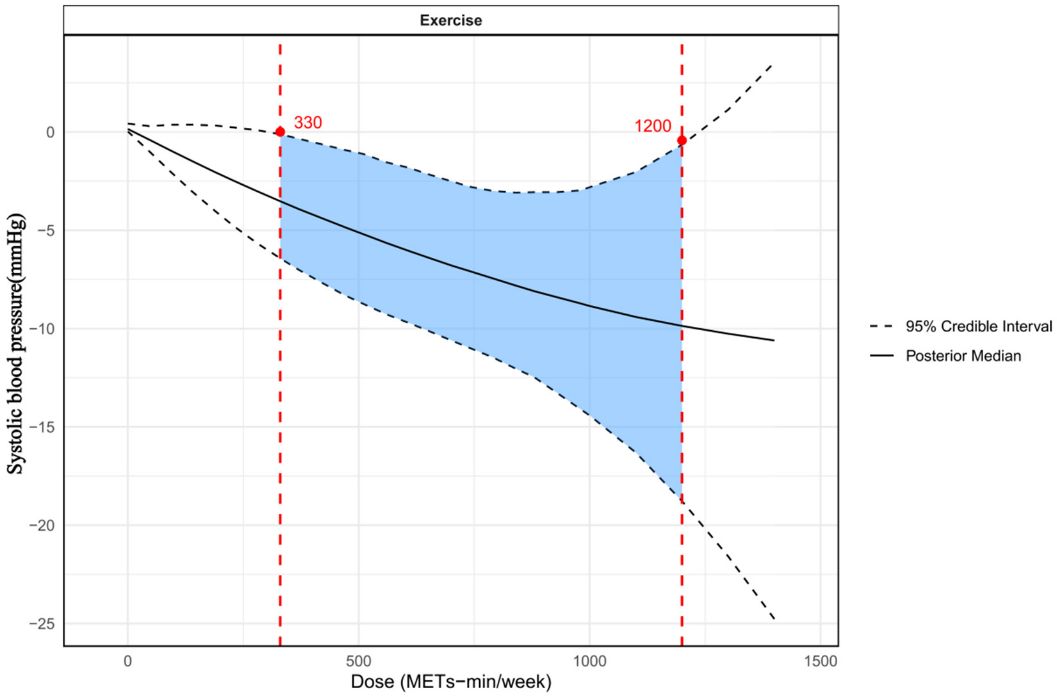

Figure S13. Dose–response relationship between exercise and systolic blood pressure (intervention length ＞12 weeks).

The red dashed lines represent the model-estimated lower and upper exposure values associated with statistically significant reductions (330 and 1200 MET-min/week), while the red shaded area indicates the statistically significant region. Red dots represent the exposure values associated with the greatest predicted reductions within the modelled range (1200 MET-min/week).

5.5.6 Systolic blood pressure (intervention length ≤12 weeks)

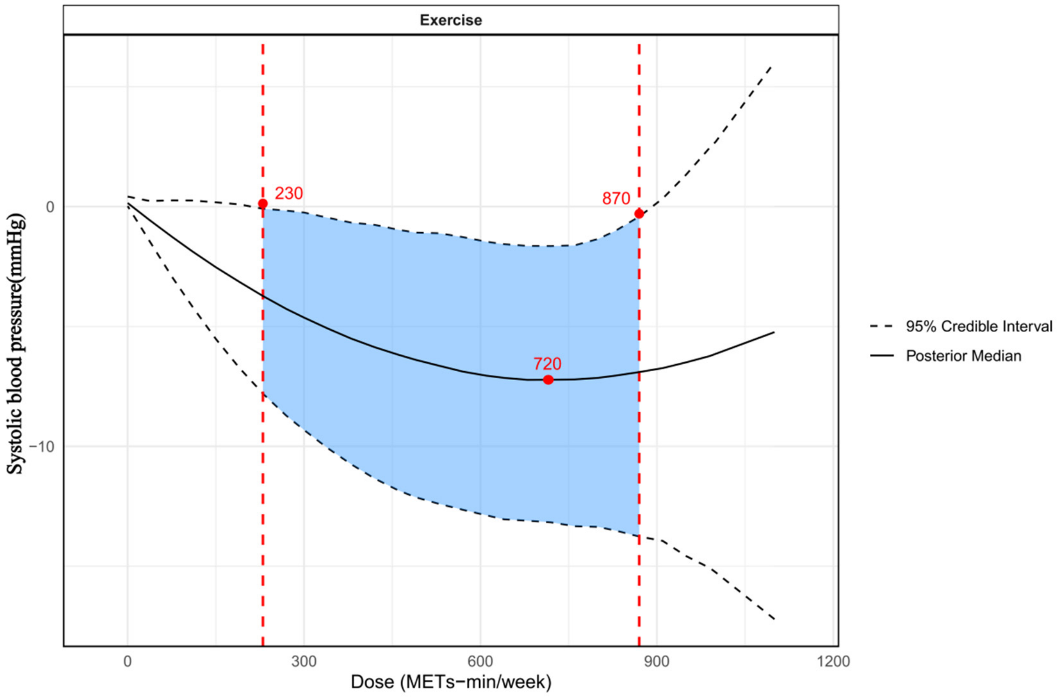

Figure S14. Dose–response relationship between exercise and systolic blood pressure (intervention length ≤12 weeks).

The red dashed lines represent the model-estimated lower and upper exposure values associated with statistically significant reductions (230 and 870 MET-min/week), while the red shaded area indicates the statistically significant region. Red dots represent the exposure values associated with the greatest predicted reductions within the modelled range (720 MET-min/week).

5.5.7 Diastolic blood pressure (baseline blood pressure ≥80 mmHg)

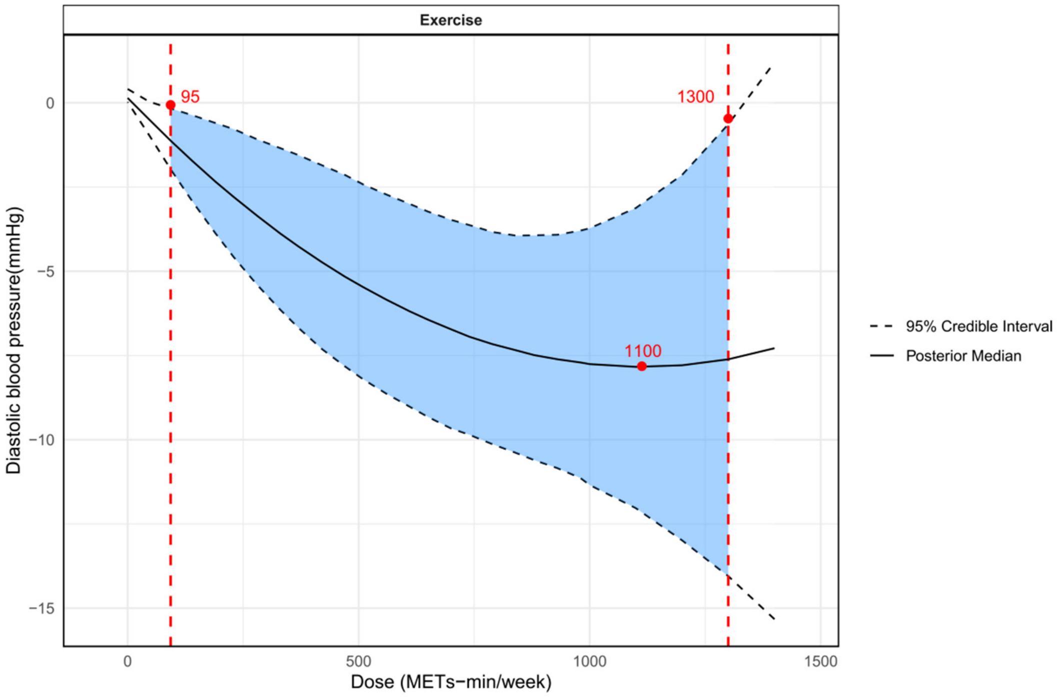

Figure S15. Dose－response relationship between exercise and diastolic blood pressure (baseline blood pressure ≥80 mmHg).

The red dashed lines represent the model-estimated lower and upper exposure values associated with statistically significant reductions (95 and 1300 MET-min/week), while the red shaded area indicates the statistically significant region. Red dots represent the exposure values associated with the greatest predicted reductions within the modelled range (1100 MET-min/week).

5.5.8 Diastolic blood pressure (baseline blood pressure <80 mmHg)

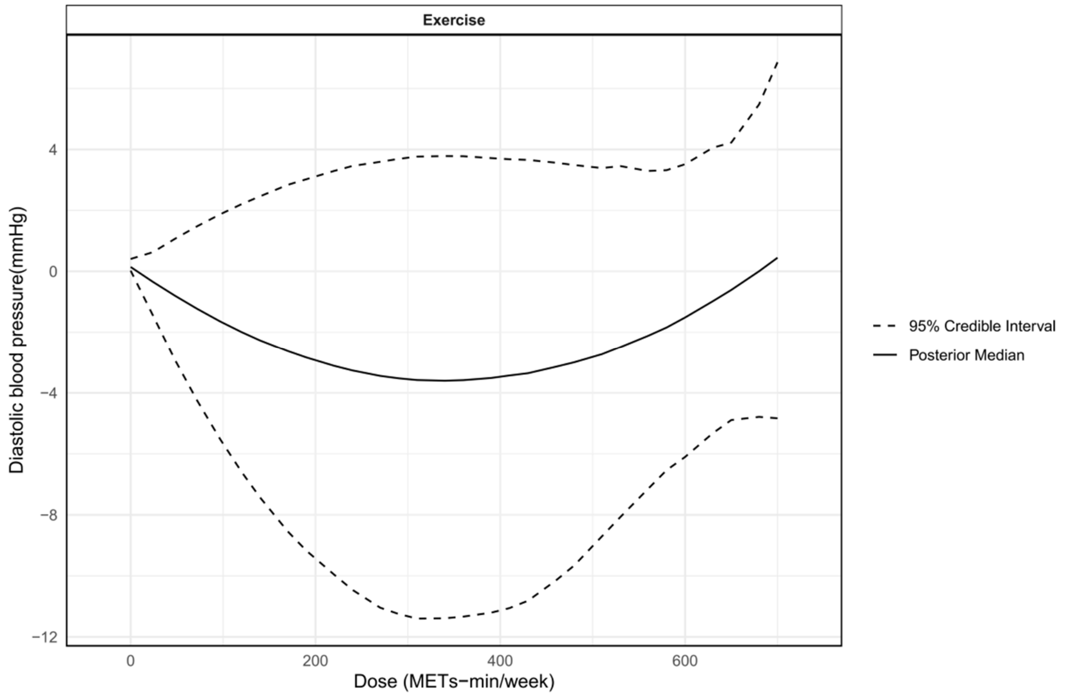

Figure S16. Dose–response relationship between exercise and diastolic blood pressure (baseline blood pressure <80 mmHg).

5.5.9 Diastolic blood pressure (non-dialysis population)

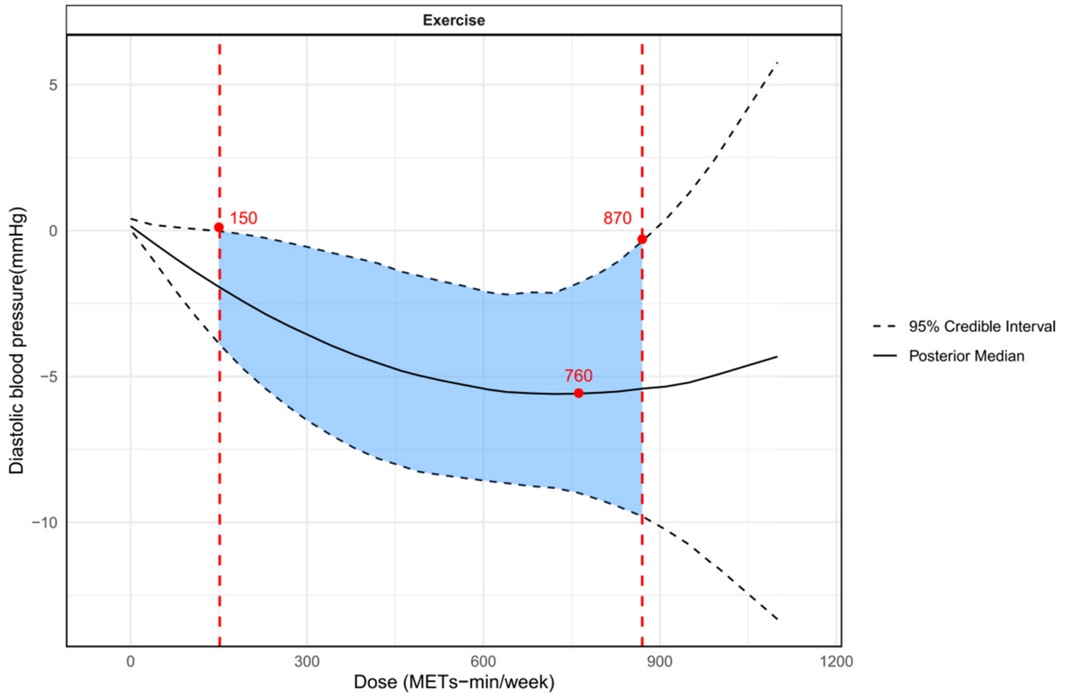

Figure S17. Dose－response relationship between exercise and diastolic blood pressure (non-dialysis population).

The red dashed lines represent the model-estimated lower and upper exposure values associated with statistically significant reductions (150 and 870 MET-min/week), while the red shaded area indicates the statistically significant region. Red dots represent the exposure values associated with the greatest predicted reductions within the modelled range (760 MET-min/week).

5.5.10 Diastolic blood pressure (dialysis population)

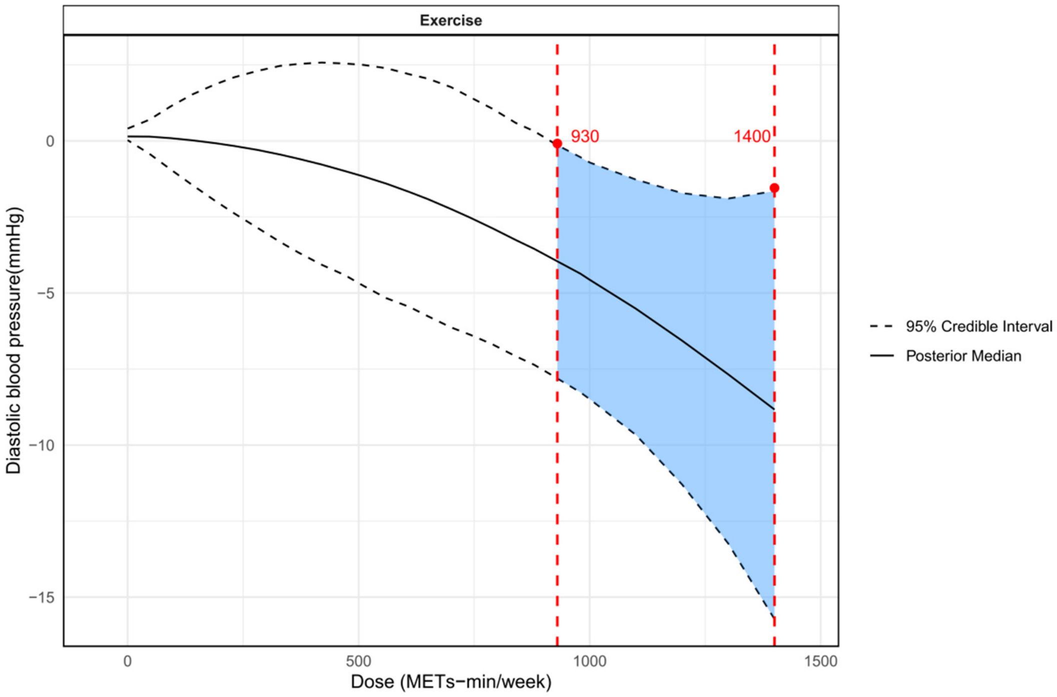

Figure S18. Dose – response relationship between exercise and diastolic blood pressure (dialysis population).

The red dashed lines represent the model-estimated lower and upper exposure values associated with statistically significant reductions (930 and 1400 MET-min/week), while the red shaded area indicates the statistically significant region. The red dot represents the exposure value associated with the greatest predicted reduction within the modelled range (1400 MET-min/week).

5.5.11 Diastolic blood pressure (intervention length ＞12 weeks)

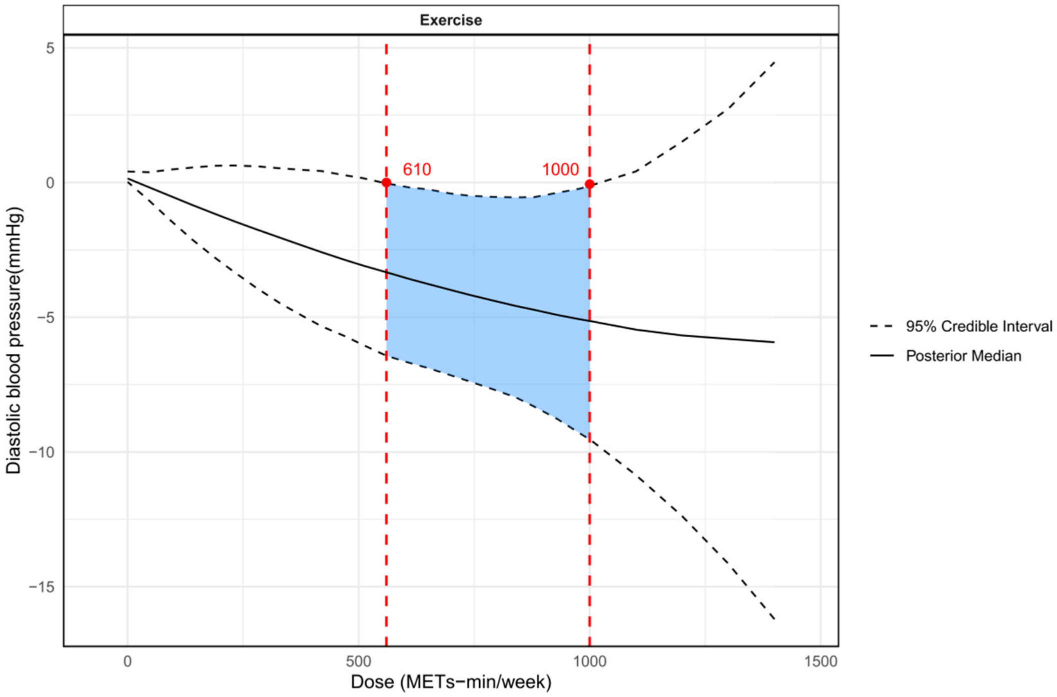

Figure S19. Dose – response relationship between exercise and diastolic blood pressure (intervention length >12 weeks).

The red dashed lines represent the model-estimated lower and upper exposure values associated with statistically significant reductions (610 and 1000 MET-min/week), while the red shaded area indicates the statistically significant region. Red dots represent the exposure values associated with the greatest predicted reductions within the modelled range (1000 MET-min/week).

5.5.12 Diastolic blood pressure (intervention length ≤12 weeks)

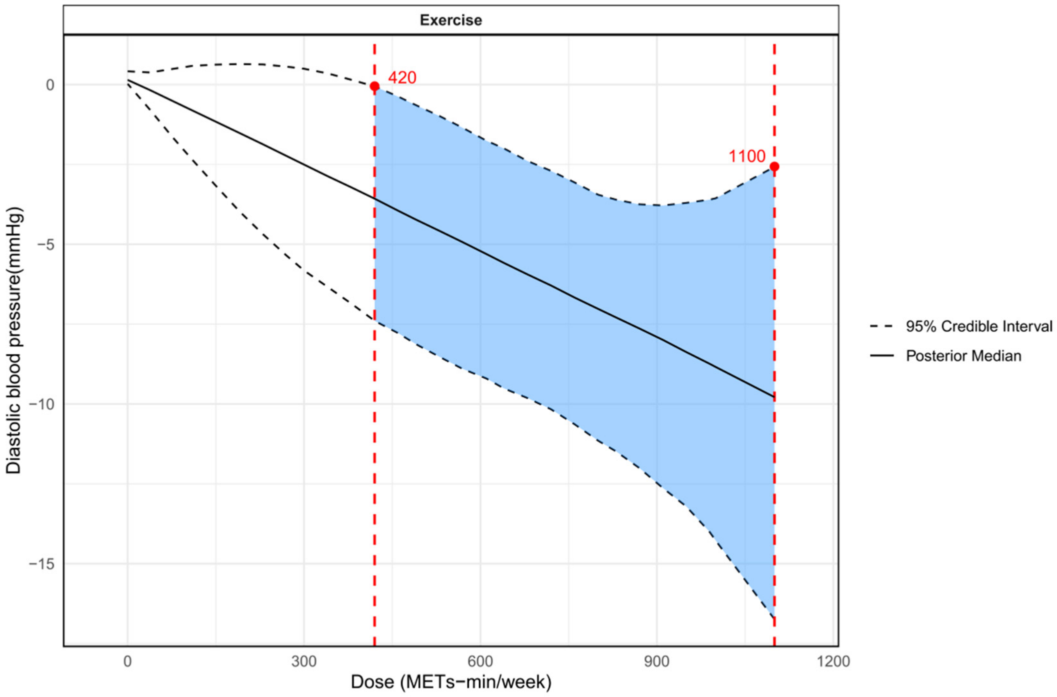

Figure S20. Dose – response relationship between exercise and diastolic blood pressure (intervention length ≤12 weeks).

The red dashed lines represent the model-estimated lower and upper exposure values associated with statistically significant reductions (420 and 1100 MET-min/week), while the red shaded area indicates the statistically significant region. Red dots represent the exposure values associated with the greatest predicted reductions within the modelled range (1000 MET-min/week).

5.6 Sensitivity analysis

5.6.1 Systolic blood pressure

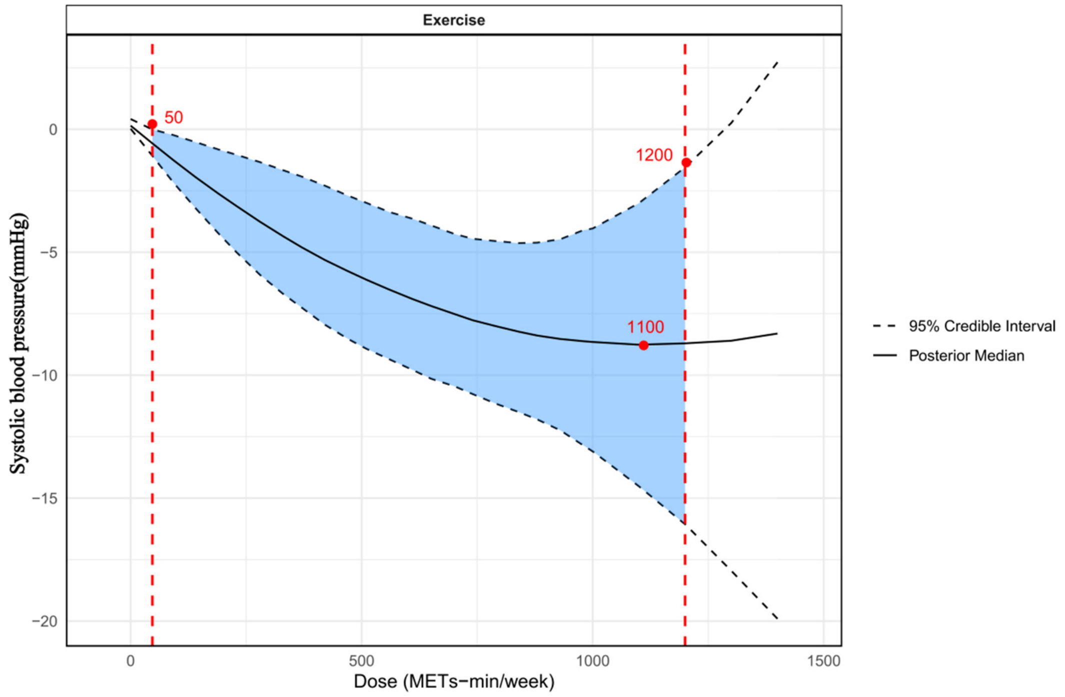

Figure S21. Dose-response relationship between exercise and systolic blood pressure in patients with CKD after excluding high-risk studies and studies with education components.

5.6.2 Diastolic blood pressure

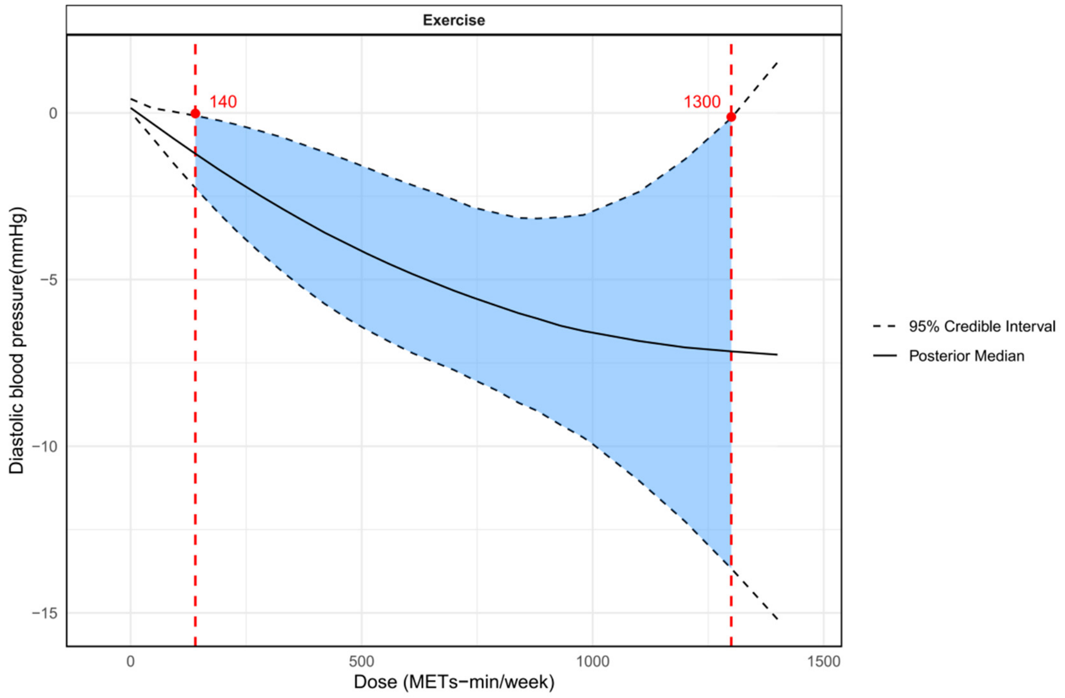

Figure S22. Dose-response relationship between exercise and diastolic blood pressure in patients with CKD after excluding high-risk studies and studies with education components.

5.7 Ranking of model-estimated exercise modalities and doses

5.7.1 Table S8. Systolic blood pressure

Rank	Treatment	Mean	Median	2.50%	97.50%
1	RT_700	11.028	4	1	53
2	RT_680	12.15333333	6	2	51
3	RT_650	13.502	9	3	50
4	Mind-body_950	14.43366667	10	1	51.025
5	Mind-body_990	14.651	10	2	55
6	Mind-body_1000	14.66733333	10	1	56.025
7	Mind-body_910	14.56133333	11	1	48
8	Mind-body_1100	16.79333333	11	1	65
9	RT_630	14.79733333	11	4	49
10	Mind-body_870	15.02433333	12	2	45
11	Mind-body_830	15.89066667	13	2	45
12	Mind-body_800	16.93666667	14	2	44
13	RT_600	16.45466667	14	5	49
14	Mind-body_760	18.38	16	3	46
15	RT_580	17.96533333	16	6	49
16	Mind-body_720	20.08133333	18	4	47
17	RT_560	19.60366667	18	7	50
18	Mind-body_680	22.072	20	6	49
19	RT_530	21.70366667	20	8	51
20	Mind-body_640	24.225	22	8	51
21	RT_510	23.541	22	9	52
22	Mind-body_610	26.20933333	24	10	53
23	RT_480	25.98666667	25	10	53
24	Mind-body_570	28.79933333	27	13	56
25	RT_460	28.07166667	27	11	55
26	Mind-body_530	31.59066667	29	14	58
27	RT_430	30.93766667	29	12	57
28	RT_410	33.31166667	31	13	59
29	Mind-body_490	34.623	32	16	62.025
30	AE_840	33.73166667	33	6.975	71
31	AE_880	34.02866667	33	6	74
32	AE_930	34.916	33	6	80.025
33	AE_790	33.817	34	8	66
34	AE_980	36.36133333	34	5	88

35	RT_390	35.80533333	34	14	61
36	AE_740	34.60566667	35	9	64
37	AE_1000	37.21933333	35	5	91
38	Mind-body_460	37.339	35	17	65
39	AE_700	35.79766667	36.5	11	63
40	AE_1100	41.382	38	4	104
41	RT_360	39.301	38	15	65
42	AE_650	37.73066667	39	13	65
43	Mind-body_420	40.894	40	18	69
44	AE_610	39.90933333	41	14	66
45	RT_340	42.10266667	42	16	68.025
46	AE_560	42.887	44	18	69
47	AE_1200	46.95066667	45	3	111
48	Mind-body_380	44.86733333	46	19	74
49	AE_510	46.26466667	47	22	73
50	RT_310	46.05333333	47	17	73
51	AE_470	49.364	50	26	75
52	RT_290	49.13266667	50	24	77
53	Mind-body_340	49.29933333	51	20	78
54	AE_1300	53.564	52	2	112
55	AE_420	53.32533333	53	31	78.025
56	RT_270	52.32566667	54	29	80
57	Mind-body_300	54.097	55.5	25	83
58	AE_370	57.54433333	57	35	82.025
59	RT_240	56.781	58	34.975	84
60	Mind-body_270	58.20766667	59	32.975	86
61	AE_330	61.33966667	60	39	85.025
62	AE_1400	60.14166667	61	1	113
63	RT_220	60.258	61	38	87
64	AE_280	66.16	64	43	90
65	Mind-body_230	63.81833333	64	39	90
66	RT_190	65.12466667	64	43	91
67	RT_170	68.86933333	67	46	93
68	AE_230	71.52466667	68	55	94
69	Mind-body_190	69.81366667	68	45	94
70	RT_140	74.408	71	55	97
71	AE_190	76.599	72	62	98
72	Mind-body_150	76.41133333	73	54.975	99
73	RT_120	78.76766667	74	61	99
74	AE-RT_470	79.83133333	79	52	99
75	AE-RT_500	79.729	79	50	100
76	AE-RT_430	80.40966667	80	55	98

77	AE-RT_530	79.93666667	80	48	101
78	AE-RT_560	80.394	80	46	102
79	AE_140	83.72133333	81	69	102
80	AE-RT_400	81.20833333	81	59	97
81	AE-RT_590	81.05966667	81	44	103
82	AE-RT_370	82.362	82	62	96
83	AE-RT_620	81.86733333	82	42	104
84	Mind-body_110	84.06066667	82	65	103
85	RT_97	84.034	83	67	103
86	AE-RT_340	83.80066667	84	65	97
87	AE-RT_650	82.863	84	40	105
88	AE-RT_310	85.45833333	86	69	98
89	AE-RT_280	87.342	87	73	100
90	AE-RT_680	83.97533333	87	38	106
91	AE-RT_710	85.16833333	89	37	107
92	AE-RT_250	89.399	90	77	102
93	AE-RT_220	91.59366667	91	80	103
94	AE-RT_740	86.45733333	92	35	108
95	RT_72	90.30466667	93	72	105
96	AE-RT_190	93.88333333	94	83	104
97	Mind-body_76	91.642	94	73	107
98	AE_93	91.85733333	95	75	106
99	AE-RT_780	88.05533333	96	33	109
100	AE-RT_160	96.28066667	97	84	106
101	AE-RT_810	89.43533333	99	31	110
102	AE-RT_120	99.28866667	101	85.975	108
103	RT_48	96.54566667	101	77	109
104	AE-RT_840	90.85433333	102	29.975	111
105	AE-RT_93	101.6676667	104	86	110
106	AE-RT_870	92.279	104	27.975	112
107	AE_47	100.5646667	105	80	111
108	Mind-body_38	100.6593333	105	80	111
109	AE-RT_62	104.3696667	107	86	111
110	RT_24	103.265	107	81	112
111	AE-RT_900	93.698	108	26	113
112	AE-RT_31	106.684	110	85	112
113	Placebo_0	108.1566667	112	84	113

Note: The number that follows the exercise intervention indicates the dose of exercise (METs-min/week). AE: Aerobic Exercise; AE-RT: Combined Aerobic and Resistance Training; RT: Resistance Training

5.7.2 Table S9. Diastolic blood pressure

Rank	Treatment	Mean	Median	2.50%	97.50%
1	RT_700	7.093333333	1	1	42.025
2	RT_680	8.28	2	2	42
3	RT_650	9.691666667	3	3	42.025
4	RT_630	10.98833333	4	4	44
5	RT_600	12.58266667	6	5	45
6	RT_580	14.01833333	8	6	46
7	Mind-body_1100	14.25133333	10	1	64.025
8	RT_560	15.51766667	10	7	46.025
9	Mind-body_1000	14.561	11	2	57
10	Mind-body_990	15.18966667	12	3	56
11	Mind-body_950	16.02533333	13	3	53.025
12	RT_530	17.41933333	13	8	48
13	Mind-body_910	17.04633333	15	4	52.025
14	RT_510	19.11066667	15	9	50
15	Mind-body_870	18.28666667	16	5	51.025
16	Mind-body_830	19.737	18	6	51
17	RT_480	21.31566667	18	10	52
18	Mind-body_800	21.171	19	7	51
19	Mind-body_760	22.93466667	21	8	53
20	RT_460	23.22866667	21	11	53
21	Mind-body_720	24.888	23	10	54
22	RT_430	25.834	24	12	56
23	Mind-body_680	27.01066667	25	11	56
24	RT_410	28.05666667	26	13	57
25	Mind-body_640	29.30033333	27	12	59
26	RT_390	30.35166667	28	14	60
27	Mind-body_610	31.40466667	29	13	62
28	Mind-body_570	34.03233333	32	14	65
29	RT_360	33.59466667	32	15	63
30	AE_1000	36.20433333	34	5	85.025
31	AE_1100	37.256	34	4	99
32	AE_1200	39.29433333	34	3	109
33	RT_340	36.288	34	16	66
34	AE_930	36.48166667	35	7	77.05
35	AE_980	36.293	35	6	82.025
36	Mind-body_530	36.85633333	35	15	68
37	AE_1300	42.111	35.5	2	112
38	AE_880	37.08333333	37	8	73
39	AE_840	37.94733333	38	9.975	71.025
40	AE_1400	45.44833333	38	1	113

41	Mind-body_490	39.87033333	38	16	72
42	RT_310	40.07566667	38	17	70
43	AE_790	39.27233333	39	11	71
44	AE_740	41.01966667	41	14	70.025
45	Mind-body_460	42.52333333	41	17	74
46	RT_290	43.148	42	18	74
47	AE_700	42.865	43	16	73
48	AE_650	45.26966667	45	19	75.025
49	Mind-body_420	45.98233333	46	18	77
50	RT_270	46.40766667	46	19	77
51	AE_610	47.608	48	22	77
52	AE_560	50.51066667	50	25	79
53	Mind-body_380	49.74066667	50	22.975	81
54	RT_240	51.033	52	26	81
55	AE_510	53.67966667	54	28	82
56	Mind-body_340	53.83866667	55	29	84
57	AE_470	56.57166667	56	31	85
58	RT_220	54.708	56	30	85
59	AE_420	60.14066667	59	35	88
60	Mind-body_300	58.32233333	59	34	87.025
61	RT_190	60.04733333	60	35	88.025
62	AE_370	63.98866667	62	39	91
63	Mind-body_270	62.13833333	62	38	90
64	RT_170	64.12266667	64	40	91
65	AE_330	67.47	65	42	94
66	Mind-body_230	67.32866667	66	42	94
67	AE_280	71.82766667	69	47.975	97
68	RT_140	70.12533333	69	45	95
69	Mind-body_190	72.893	70	47	97
70	RT_120	74.75633333	72	49	98
71	AE_230	76.75	73	57	100
72	Mind-body_150	78.97533333	75	56	100
73	RT_97	80.261	76	56	101
74	AE_190	81.201	77	63	102
75	AE-RT_560	80.763	80	46	102
76	AE-RT_590	80.48566667	80	44	103
77	AE-RT_620	80.33566667	80	41	104
78	AE-RT_650	80.31366667	80	38	105
79	AE-RT_500	81.77533333	81	52	101.025
80	AE-RT_530	81.17966667	81	49	101
81	AE-RT_680	80.41233333	81	36	106
82	AE-RT_710	80.606	81	34	107

83	AE-RT_470	82.51333333	82	54	101.025
84	AE-RT_740	80.92633333	82	31	108
85	AE-RT_430	83.652	84	58	102
86	AE-RT_780	81.368	84	28	109
87	AE-RT_400	84.747	85	61	103
88	AE-RT_810	81.826	85	25	110
89	AE-RT_370	86.00933333	86	63	104
90	Mind-body_110	85.76666667	86	65	104
91	AE-RT_840	82.35466667	87	23	111
92	AE-RT_340	87.40266667	88	66	104
93	RT_72	86.676	88	64	105
94	AE_140	87.23	89	69	105
95	AE-RT_870	82.917	89	21	112
96	AE-RT_310	88.913	90	69	105
97	AE-RT_900	83.561	91	19	113
98	AE-RT_250	92.28733333	92	76	106
99	AE-RT_280	90.54066667	92	73	105
100	AE-RT_220	94.10133333	95	80	106
101	Mind-body_76	92.161	96	72	107
102	AE-RT_190	95.96266667	97	83	107
103	AE_93	93.629	98	75	108
104	RT_48	93.34833333	98	71	108
105	AE-RT_160	97.85366667	100	84	108
106	AE-RT_120	100.2346667	103	84	109
107	AE-RT_93	102.105	106	85	110
108	Mind-body_38	99.94533333	106	79	111
109	RT_24	100.601	106	79	111
110	AE_47	100.6523333	107	80	111
111	AE-RT_62	104.18	108	85.975	111
112	AE-RT_31	105.9506667	112	85	112
113	Placebo_0	107.054	113	84	113

Note: The number that follows the exercise intervention indicates the dose of exercise (METs-min/week). AE: Aerobic Exercise; AE-RT: Combined Aerobic and Resistance Training; RT: Resistance Training

5.8 Meta-regression

5.8.1 Systolic blood pressure

Table S10. Dose-response network meta-regression (systolic blood pressure)

Covariate	DIC	ResidualDeviance	Systolic blood pressure<br>Shared beta<br>(median and 95%CrI)	SD
Age (years)	351.6	62.1	0.43 (-0.26, 1.41)	0.78 (0.12, 2.14)
Baseline blood pressure (mm Hg)	338.7	53.9	-0.04<br>(-0.76, 0.57)	0.72 (0.28, 1.56)
Intervention length (weeks)	309.6	73	0.049<br>(-0.16, 0.23)	0.14(0.005, 0.57)

Note: DIC: deviance information criterion; SD: standard deviation

5.8.2 Diastolic blood pressure

Table S11. Dose-response network meta-regression (diastolic blood pressure)

Covariate	DIC	ResidualDeviance	Diastolic blood pressure<br>Shared beta<br>(median and 95%CrI)	SD
Age (years)	342.3	73.3	-0.09 (-0.87, 0.75)	0.82 (0.036, 2.41)
Baseline blood pressure (mm Hg)	277	53.9	-0.15<br>(-0.7, 0.45)	0.56 (0.29, 1.16)
Intervention length (weeks)	300.9	80	0.11(-0.01, 0.24)	0.11 (0.01, 0.35)

Note: DIC: deviance information criterion; SD: standard deviation

6. Publication bias

6.1 Systolic blood pressure

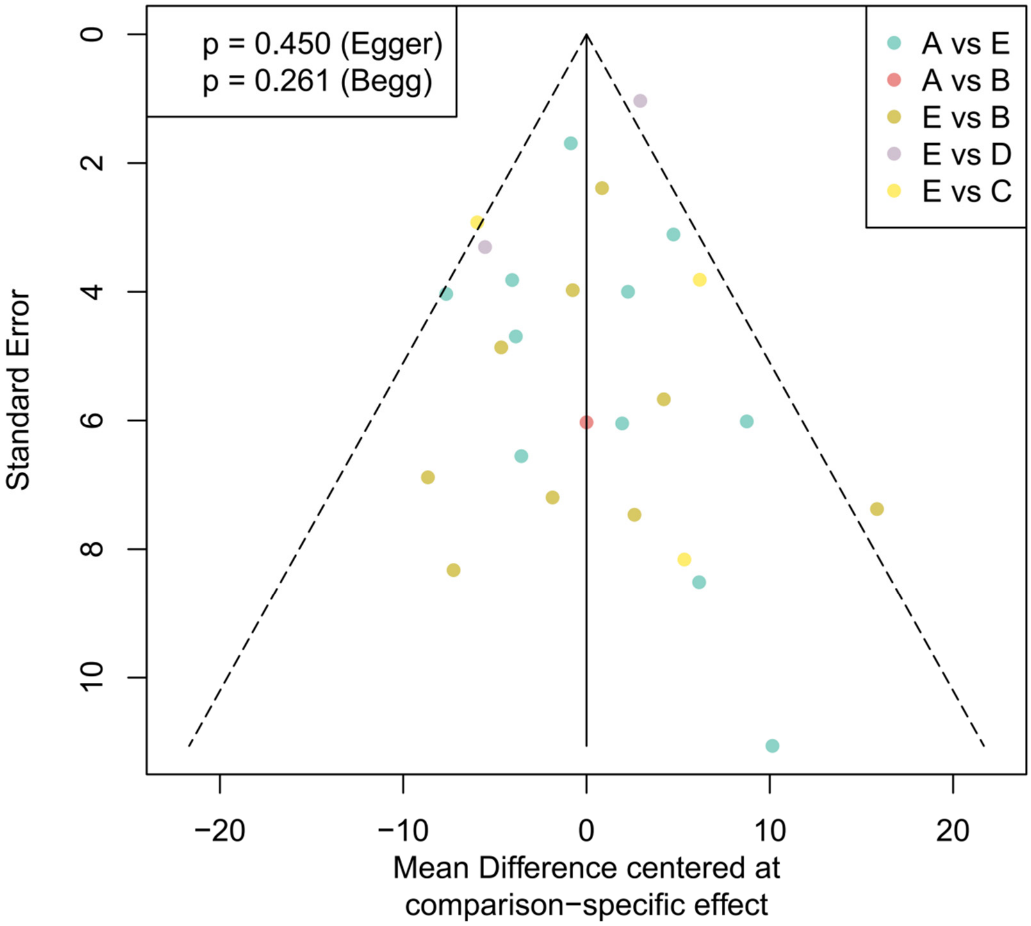

Figure S23. funnel plot (systolic blood pressure).

A: aerobic exercise; B: Combined Aerobic and Resistance Training; C:Mind-body exercise; D: Resistance Training; E:Control group

6.2 Diastolic blood pressure

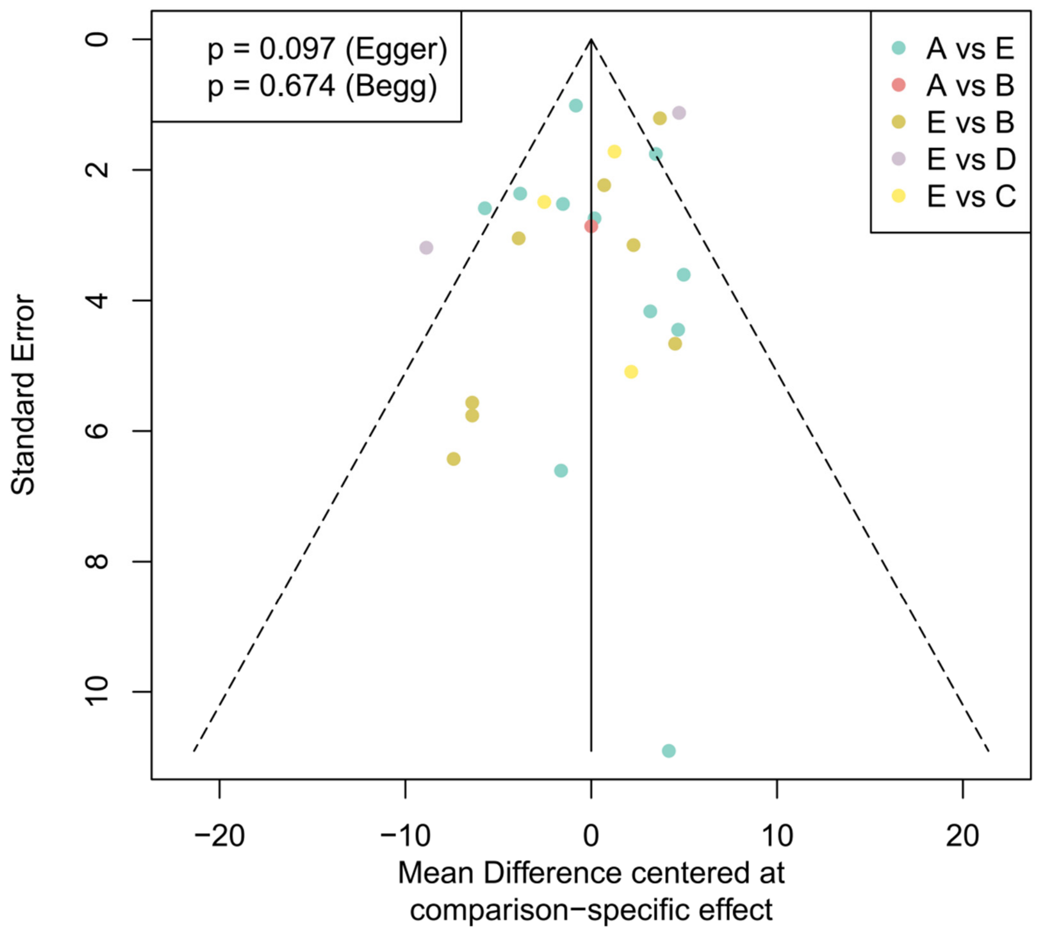

Figure S24. funnel plot (diastolic blood pressure).

A: aerobic exercise; B: Combined Aerobic and Resistance Training; C:Mind-body exercise; D: Resistance Training; E:Control group

7. Grade summary of all studies

Based on the CINeMA online guidelines (https://cinema.ispm.unibe.ch/), we evaluated blood pressure only. Downgrading decisions were made based on the following criteria:

7.1 Within-study Bias

Each included study was rated for risk of bias as low (RoB2 1 point), moderate (RoB2 2 points), or high (RoB2 3 points). The rule applied was the average RoB. No downgrade occurred for “no concerns”; one level was downgraded for “some concerns,” and two levels for “major concerns.”

7.2 Reporting Bias (Publication Bias and Selective Reporting)

To minimize publication bias, we examined the reference lists of recent relevant studies. Publication bias was assessed using comparison-adjusted Funnel plots and Begg's and

Egger's tests (Supplementary Content 6). The results indicated no statistically significant small-study effects ($p > 0.05$), suggesting no clear evidence of publication bias. Furthermore, no high risk of selective reporting was identified; therefore, this domain was not downgraded.

7.3 Indirectness

Indirectness was assessed by examining the clinical and methodological relevance of the included studies to the research question. All studies fulfilled the PICOS criteria, targeting adults with CKD, comparing exercise interventions with appropriate controls, and reporting systolic and/or diastolic blood pressure outcomes. Variation in CKD stage, dialysis status, comorbidities, and intervention characteristics was considered under indirectness and heterogeneity when judging confidence in the evidence.

7.4 Imprecision

Blood pressure outcomes were treated as continuous variables. The mean difference was used as the effect size (change score: endpoint minus baseline) because blood pressure was measured in consistent units across studies. Because no single minimal clinically important difference is universally accepted across CKD and dialysis populations for exercise trials, the null value (0) was used as the reference threshold for interpreting statistical significance.

7.5 Heterogeneity

In addition to statistical heterogeneity, the clinical heterogeneity caused by the widespread distribution of chronic kidney disease (CKD) subtypes and baseline blood pressure differences reduced the heterogeneity scores of all intervention models by two levels.

7.6 Incoherence

Incoherence was addressed by p-value thresholds: no downgrade for $p > 0.1$, one level for p 0.05–0.1, and two levels for $p < 0.05$.

7.7 Summarizing Judgments Across CINeMA Domains

The CINeMA framework assesses confidence in evidence across six domains: within-study bias, reporting bias, indirectness, imprecision, heterogeneity, and incoherence. These judgments are summarized into an overall confidence rating using the GRADE approach (high, moderate, low, or very low). As a single issue may impact multiple domains (e.g., indirectness due to intransitivity or heterogeneity affecting both imprecision and bias), all domains are considered together to avoid redundant downgrading and ensure a balanced assessment.

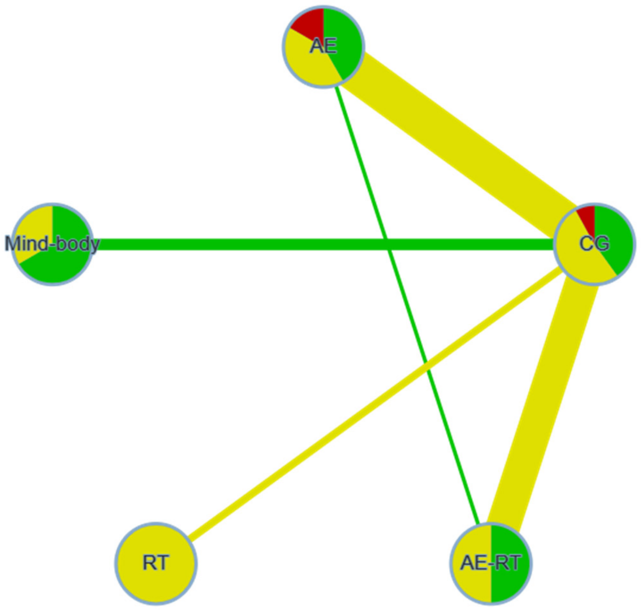

Figure S25. Network plot of study limitations of the included studies (systolic blood pressure).

The colors in the circles indicate the percentage of low RoB studies (green), moderate RoB studies (yellow), high RoB studies (red) about each physical activity type. Edge width by the number of studies. Edge color by average RoB. The colors of the lines indicate the summative RoB assessment of each comparison. Low RoB is green, moderate RoB is yellow, high RoB is red.AE: Aerobic Exercise; AE-RT: Combined Aerobic and Resistance Training; RT: Resistance Training; Mind-body: Mind-body exercise

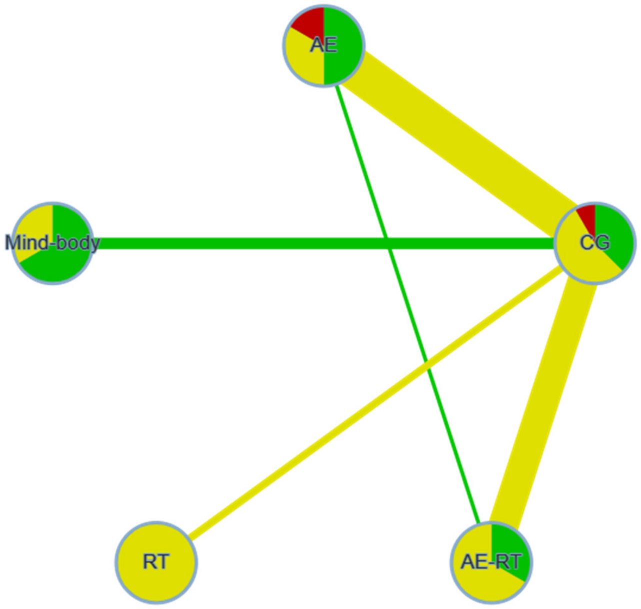

Figure S26. Network plot of study limitations of the included studies (diastolic blood pressure).

The colors in the circles indicate the percentage of low RoB studies (green), moderate RoB studies (yellow), high RoB studies (red) about each physical activity type. Edge width by the number of studies. Edge color by average RoB. The colors of the lines indicate the summative RoB assessment of each comparison. Low RoB is green, moderate RoB is yellow, high RoB is red. AE: Aerobic Exercise; AE-RT: Combined Aerobic and Resistance Training; RT: Resistance Training

Table S12. The confidence of evidence using the CINeMA (systolic blood pressure).

Comparison	Within-study bias	Reporting bias	Indirectness	Imprecision	Heterogeneity	Incoherence	Confidence rating	Reason(s) for downgrading
AE:AE-RT	Some concerns	Low risk	No concerns	Major concerns	No concerns	No concerns	**Very low**	["Within-study bias","Imprecision"]
**AE:CG**	**Some concerns**	**Low risk**	**No concerns**	**No concerns**	**Major concerns**	**No concerns**	**Very low**	**["Within-study bias","Heterogeneity"]**
**AE-RT:CG**	**No concerns**	**Low risk**	**No concerns**	**Major concerns**	**No concerns**	**No concerns**	**Low**	**["Imprecision"]**
**Mind-body:CG**	**No concerns**	**Low risk**	**No concerns**	**No concerns**	**No concerns**	**No concerns**	**High**	**[]**
**RT:CG**	**Some concerns**	**Low risk**	**No concerns**	**No concerns**	**No concerns**	**No concerns**	**Moderate**	**["Within-study bias"]**

AE: Aerobic Exercise; AE-RT: Combined Aerobic and Resistance Training; RT: Resistance Training;Mind-body: Mind-body exercise

Table S13. The confidence of evidence using the CINeMA (diastolic blood pressure).

Comparison	Number of studies	Within-study bias	Reporting bias	Indirectness	Imprecision	Heterogeneity	Incoherence	Confidence rating	Reason(s) for downgrading
AE:AE-RT	1	Some concerns	Low risk	No concerns	Major concerns	No concerns	No concerns	Very low	["Within-study bias","Imprecision"]
**AE:CG**	**11**	**Some concerns**	**Low risk**	**No concerns**	**No concerns**	**Major concerns**	**No concerns**	**Very low**	**["Within-study bias","Heterogeneity"]**
**AE-RT:CG**	**8**	**Some concerns**	**Low risk**	**No concerns**	**Major concerns**	**No concerns**	**No concerns**	**Very low**	**["Within-study bias","Imprecision"]**
**Mind-body:CG**	**3**	**No concerns**	**Low risk**	**No concerns**	**No concerns**	**No concerns**	**No concerns**	**High**	**[]**
**RT:CG**	**2**	**Some concerns**	**Low risk**	**No concerns**	**No concerns**	**No concerns**	**No concerns**	**Moderate**	**["Within-study bias"]**

AE: Aerobic Exercise; AE-RT: Combined Aerobic and Resistance Training; RT: Resistance Training;Mind-body: Mind-body exercise

Table S14. Summary of dose-response network meta-analysis result.

Category	Data point	Blood pressure	Model-estimated statistically significant range (MET-min/week)	Exposure associated with greatest model-estimated reduction (MET-min/week)	Posterior median MD and 95% CrI at that exposure
Overall exercise	56	SBP	95-1200	1200	-8.53 mm Hg (-15.43，-1.59)
	54	DBP	190-1300	1300	-7.30 mm Hg (-14.17，-0.68)
Baseline SBP (≥140 mmHg)	23	SBP	230-1200	1100	-8.70 mm Hg (-15.77，-2.27)
Baseline SBP (<140 mmHg)	19	SBP	60-530	370	-7.43 mm Hg (-12.75，-0.90)
Baseline DBP (≥80 mmHg)	33	DBP	95-1300	1100	-7.77 mm Hg (-12.03，-3.12)
Baseline DBP (<80 mmHg)	10	DBP	NA	NA	NA
Dialysis patients	18	SBP	560-1300	1300	-13.73 mm Hg (-25.97，-1.18)
Non-dialysis patients	38	SBP	75-830	570	-6.40 mm Hg (-9.73，-2.63)
Dialysis patients	18	DBP	930-1400	1400	-8.80 mm Hg (-15.72，-1.65)
Non-dialysis patients	36	DBP	150-870	760	-5.57 mm Hg (-8.98，-1.82)
Length (＞12 weeks)	40	SBP	330-1200	1200	-9.81 mm Hg (-18.77，-0.65)
Length (≤12 weeks)	16	SBP	230-870	720	-7.26 mm Hg (-13.17，-1.65)
Length (＞12 weeks)	38	DBP	610-1000	1000	-5.04 mm Hg (-9.28,-0.45)
Length (≤12 weeks)	16	DBP	420-1100	1100	-9.74 mm Hg (-16.74,-2.58)
Sensitivity analysis	51	SBP	50-1200	1100	-8.73 mm Hg (-14.51，-3.01)
	49	DBP	140-1300	1300	-7.14 mm Hg (-13.67，-0.16)
AE	23	SBP	45-1100	880	-7.73 mm Hg (-12.85，-2.67)
	20	DBP	190-1100	1100	-5.61 mm Hg (-10.81，-0.37)
AE-RT	20	SBP	NA	NA	NA
	18	DBP	NA	NA	NA
Mind-body exercise	28	SBP	40-1100	1000	-11.31 mm Hg (-19.28，-3.91)
	27	DBP	75-1100	1100	-9.02 mm Hg (-15.48，-2.37)
RT	29	SBP	25-700	700	-11.86 mm Hg (-18.08，-4.31)
	28	DBP	50-700	700	-10.68 mm Hg (-16.98，-3.58)

Table S15. Model-derived exercise accumulation estimates for blood pressure reduction.

Blood pressure	Exercise modality	Assigned MET value (Compendium code)[a]	Model-derived range (min/week)	Minutes/week at greatest model-estimated reduction	Certainty of evidence (grade)
Systolic blood pressure	Cycling	7 (code: 01016)[b]	5-155	125	**Very low**
	Jogging	4.8(code:12025)[c]	10-230	185	**Very low**
	Walking	3.5(code:17160)[d]	15-315	250	**Very low**
	Resistance training	3.5(code:02054)[e]	5-200	200	**Moderate**
	Yoga	2.3(code:02175)	15-480	435	**High**
	Tai Chi	3.3(code:15670)	10-335	305	**High**
Diastolic blood pressure	Cycling	7 (code: 01016)[b]	25-155	155	**Very low**
	Jogging	4.8(code:12025)[c]	40-230	230	**Very low**
	Walking	3.5(code:17160)[d]	55-315	315	**Very low**
	Resistance training	3.5(code:02054)[e]	15-200	200	**Moderate**
	Yoga	2.3(code:02175)	30-480	480	**High**
	Tai Chi	3.3(code:15670)	20-335	335	**High**

Note: [a]Exercise intensity was coded based on the 2024 standardized physical activity intensity tables. [b]Cycling: self-selected moderate pace. [c]Jogging, in place. [d]Walking for pleasure. [e]Resistance (weight) training, multiple exercises, 8-15 reps at varied resistance. 3-6 METs represents moderate-intensity activity, and >6 METs represents high-intensity activity, serving as a reference for absolute exercise intensity. METs-min: Metabolic Equivalents of Task minute
